# Endogenous small intestinal microbiome determinants of transient colonisation efficiency by bacteria from fermented dairy products: a randomised controlled trial

**DOI:** 10.1186/s40168-023-01491-4

**Published:** 2023-03-07

**Authors:** Edoardo Zaccaria, Tim Klaassen, Annick M. E. Alleleyn, Jos Boekhorst, Tamara Smokvina, Michiel Kleerebezem, Freddy J. Troost

**Affiliations:** 1 Host Microbe Interactomics Group, Wageningen University & Research, De Elst 1, 6708WD Wageningen, The Netherlands; 2 Food Innovation and Health, Center for Healthy Eating and Food Innovation, Maastricht University, Venlo, 5911AA The Netherlands; 3 Division of Gastroenterology-Hepatology, Department of Internal Medicine, School of Nutrition and Translational Research in Metabolism (NUTRIM), Maastricht University Medical Center+, P.O. Box 5800, 6202AZ Maastricht, The Netherlands; 4 Danone Nutricia Research, Av. De la Vauve, 91767 Palaiseau, France

**Keywords:** Small intestine, Fermented products, Personalised microbiome, Human trial

## Abstract

**Background:**

The effects of fermented food consumption on the small intestine microbiome and its role on host homeostasis are largely uncharacterised as our knowledge on intestinal microbiota relies mainly on faecal samples analysis. We investigated changes in small intestinal microbial composition and functionality, short chain fatty acid (SCFA) profiles, and on gastro-intestinal (GI) permeability in ileostomy subjects upon the consumption of fermented milk products.

**Results:**

We report the results from a randomised, cross-over, explorative study where 16 ileostomy subjects underwent 3, 2-week intervention periods. In each period, they consumed either milk fermented by *Lacticaseibacillus rhamnosus* CNCM I-3690, or milk fermented by *Streptococcus thermophilus* CNCM I-1630 and *Lactobacillus delbrueckii* subsp. *bulgaricus* CNCM I-1519, or a chemically acidified milk (placebo) daily. We performed metataxonomic, metatranscriptomic analysis, and SCFA profiling of ileostomy effluents as well as a sugar permeability test to investigate the microbiome impact of these interventions and their potential effect on mucosal barrier function. Consumption of the intervention products impacted the overall small intestinal microbiome composition and functionality, mainly due to the introduction of the product-derived bacteria that reach in several samples 50% of the total microbial community. The interventions did not affect the SCFA levels in ileostoma effluent, or gastro-intestinal permeability and the effects on the endogenous microbial community were negligible. The impact on microbiome composition was highly personalised, and we identified the poorly characterised bacterial family, *Peptostreptococcaceae*, to be positively associated with a low abundance of the ingested bacteria. Activity profiling of the microbiota revealed that carbon- versus amino acid-derived energy metabolism of the endogenous microbiome could be responsible for the individual-specific intervention effects on the small intestine microbiome composition and function, reflected also on urine microbial metabolites generated through proteolytic fermentation.

**Conclusions:**

The ingested bacteria are the main drivers of the intervention effect on the small intestinal microbiota composition. Their transient abundance level is highly personalised and influenced by the energy metabolism of the ecosystem that is reflected by its microbial composition (http://www.clinicaltrials.gov, ID NCT NCT02920294).

Video Abstract

**Supplementary Information:**

The online version contains supplementary material available at 10.1186/s40168-023-01491-4.

## Introduction

The human body hosts a multitude of microbial communities that occupy different body niches forming complex host-microbial ecosystems. There is increasing evidence of the importance of these microbial communities for host health and homeostasis [1, 2], with a particular focus on the colon microbiome [3]. However, the small intestine (SI) is not only the most prominent region for nutrient digestion and absorption [4] but also the intestinal region where food, bacteria and mucosa are in relatively close contact. This creates an environment of prominent microbiota interactions with the diet and the host mucosa [5] that can be expected to be very important for human health [6]. This is exemplified by recent rodent studies that established prominent roles of the SI microbiota in whole body glucose homeostasis [7, 8], lipid digestion and absorption [9], and bile acid metabolism [10]. Moreover, the SI microbiota produces several important micronutrients like vitamin K and B12 [11, 12], as well as other metabolites, such as short chain fatty acids (SCFA) that can be sensed and processed by humans and thereby affect human health.

Contrary to the colonic microbiota, the human SI microbiota is poorly characterised, due to the invasive sampling technologies required to obtain material from the SI tract in healthy subjects. This can in part be overcome by sampling from individuals who underwent colectomy to remedy diseases like colorectal cancer, ulcerative colitis, or Crohn’s disease. In some of these surgical interventions the ileum is connected to a stoma in their abdominal wall (ileostoma), allowing non-invasive sampling from the SI tract. Despite their history of intestinal disease, and provided that comorbidities are absent, these ileostomists do not need maintenance medication and their SI is considered to function similarly as that of a healthy individual [13]. This is supported by the similar microbiota composition encountered in ileostoma effluent samples compared to that in SI samples obtained from healthy subjects [14, 15].

Most of the SI is a harsh environment for bacteria due to high concentrations of digestive enzymes, bile salts, and antimicrobial peptides. Microbial density and diversity are relatively low [15, 16], ranging from approximately 10^4–5^ to 10^7–9^ cells per mL intestinal content in the duodenum and distal ileum, respectively [16]. The SI microbiota is predominantly composed of facultative anaerobic bacterial taxa [17], displaying higher compositional dynamics compared to the colon due to rapid responses to the changing nutrient availability [14]. Metagenomic and metatranscriptomic studies corroborated the dynamic nature of the SI microbiota that comprises a broad repertoire of catabolic pathways for simple sugars, rather than complex carbohydrates that are enriched in the colonic microbiota [14, 15]. These findings have started to shed light on the human small intestinal microbiome and pave the way towards a better understanding of its role in health and disease.

Several food products are rich in viable bacteria that may transiently dominate the SI ecosystem. For example, yogurt is a commonly consumed fermented dairy product containing more than 10^9^ viable bacteria of *Lactobacillus delbrueckii* subspecies *bulgaricus* (*L. bulgaricus*) and *Streptococcus thermophilus* per millilitres. Likewise, probiotics that are defined as “live microorganisms that, when consumed in adequate amounts, confer a health benefit on the host” [18], are typically consumed at 10^8–12^ viable bacteria per serving. Nevertheless, only few studies have investigated the effect of live bacteria ingestion on the small intestinal microbial population composition and function [19–21]. Products containing probiotics have been shown to deliver beneficial effects on gut functions in randomised controlled studies [22], and commonly contain strains belonging to the genera *Bifidobacterium* or the bacteria formerly known as species within the genus *Lactobacillus*, which has recently been taxonomically reclassified into 23 novel genera [23]. Among the latter, some strains of *Lacticaseibacillus rhamnosus* are considered potential probiotics, and strain-specific relief of symptoms in diseases associated with intestinal microbiota dysbiosis and maintenance of the gut homeostasis have been reported [24–26]. Specifically, preclinical studies underlined the role of *L. rhamnosus* CNCM I-3690 in restoration of impaired intestinal barrier functions through its anti-inflammatory effect [27, 28], reduction of inflammation in a murine model for colitis [29] and suppression of the immune and metabolic impairments caused by the pathobiont *Bilophila wadsworthia* [30]. *In addition, the strain* supported several other health benefits like limiting weight gain, improving glucose-insulin homeostasis, and hepatic steatosis [31]. Moreover, a recent human randomised controlled trial established the safety of *L. rhamnosus* CNCM I-3690 consumption, and reported on its persistence in the human digestive track and the limited impact on faecal microbiota when combined in a multi-strain fermented milk product [32]. These preclinical findings underpin the interest in this strain as a probiotic candidate.

The aim of the present study was to investigate how fermented dairy products could (transiently) impact the small intestinal microbiota in human subjects. To this end, 16 ileostomists were recruited that underwent 3 randomised, cross-over interventions of 2 weeks, during which they consumed dairy products fermented with *Lacticaseibacillus rhamnosus* CNCM I-3690, or yogurt that was produced using *S. thermophilus* CNCM I-1630 and *L*. *bulgaricus* CNCM I-1519, or a chemically-acidified milk product that served as a placebo. Ileostoma effluent samples were collected at regular intervals during these interventions and the intermittent wash-out periods (2 weeks) for microbiota composition analysis. In addition, at the start and end of each intervention period, SI microbiota activity was assessed by metatranscriptomic analysis, and SI mucosal permeability and short-chain fatty acid levels in the stoma effluent were determined.

## Materials and methods

### Subjects

Adult ileostomy patients without comorbidities were recruited for this interventions study, using specific in- and exclusion criteria (see Supplemental methods “recruitment criteria”). Importantly, to avoid confounding effects on the study endpoints, study subjects were not allowed to consume pro-, pre- or symbiotics, fresh dairy fermented products (such as yogurt, cottage cheese, buttermilk, or soft-raw cheeses) as well as other food products that are fermented (i.e. sauerkraut) during the study period and three months prior to participation in this trial (a list of forbidden products was provided).

Due to a lack of reliable prior art in the investigation of the impact of food-derived bacteria on the small intestinal microbiota, no effect size could be estimated or a priori power calculation could be performed. Therefore, the study was designed as an explorative study, aiming for the inclusion of 15 to 20 subjects fulfilling the inclusion criteria. We recruited 15 subjects with a standard ileostoma, and a single subject with a continent-ileostoma (i.e. Kock pouch). During data analysis, the SI microbiota of the latter individual was drastically different from the rest of the ileostomists and was therefore considered to be a biological outlier that was excluded from further analyses (see “Results” section).

### Study design

**Fig. 1:**
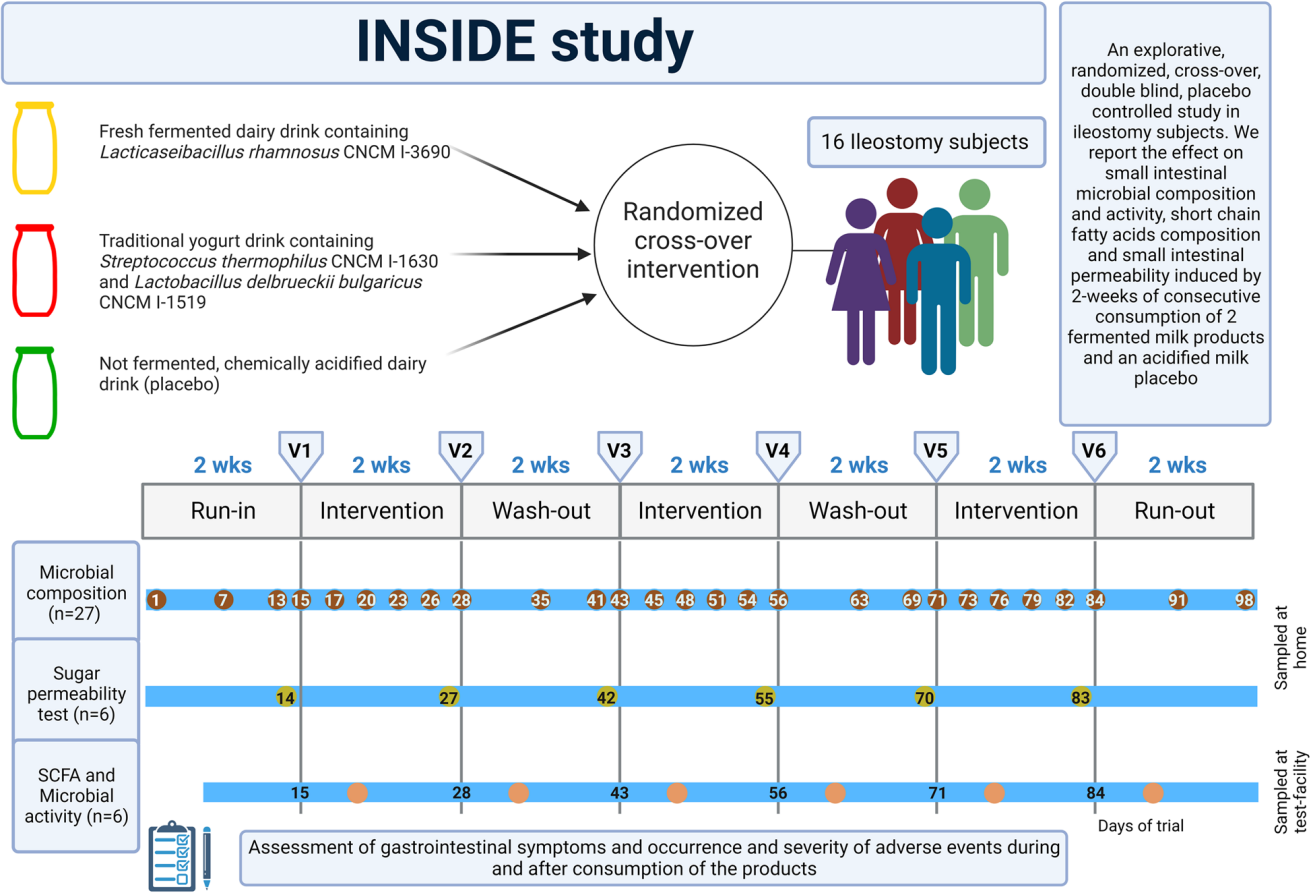
Study design. All the subjects participated in 3 intervention periods, each encompassing 14 consecutive days (± 1 day) separated by a 2-week wash-out period and preceded by a run-in period of 14 days and followed by a 14-day run-out period. Three sampling schemes overlay the timeline of the study. Microbial composition scheme indicates the days at which subjects collected ileal effluent samples at home. SCFA and microbial activity indicates the days at which subjects will participate in test days, where they filled in VAS scales to assess gastrointestinal symptoms, received standardised meals and collected urine and ileal effluent samples. Sugar permeability test scheme indicates the days at which intestinal permeability was assessed

In this randomised, double-blinded placebo-controlled crossover study, ileostomy patients consecutively received three different interventions (one serving per day, at breakfast 100 ml of intervention product) for 14 consecutive days each (± 1 day) with a 2-week wash-out in between. Moreover, the study started with a 2-week run-in period and ended with a 2-week run-out period. In both periods, study subjects did not follow a specific diet a part from to not be allowed to consumed products that could influence the study (described above). At the first and last day of each intervention period (referred to as “test days” and labelled V1–V2, V3–V4, and V5–V6 for the first, second and third intervention respectively), subjects were asked to visit the testing facility (Fig. 1). Prior to the start of the study, an external provider, AtlanStat, generated the randomisation list used for the succession of product intervention (6 sequences of 3 interventions, balance on 20 subjects, block size: 6). The list was forwarded to the person responsible for preparation of study products in Danone for the purpose of labelling.

All intervention products were manufactured by Danone (Danone Nutricia Research, Palaiseau, France) with the intention to minimise their distinction on basis of appearance and taste, including the use of similar packaging and the use of the same flavour compounds, and fully blinded. Intervention products were 100 mL of milk fermented by *Lacticaseibacillus rhamnosus* CNCM I-3690 (*L. rhamnosus*) at approximately 1–5 × 10^9^ CFU/mL (i.e., daily intake~ 1–5 × 10^11^ CFU), milk fermented by the yogurt symbionts *Streptococcus thermophilus* CNCM I-1630 and *Lactobacillus delbrueckii* subsp*. bulgaricus* CNCM I-1519 (yogurt) at approximately 10^5^-10^6^ CFU/mL for *L. bulgaricus* and 5 × 10^7^ − 10^9^ CFU/mL for *S. thermophilus* (i.e. daily intake ~ 10^7^−10^8^ and ~ 5 × 10^9^ − 10^11^ CFU, respectively), and a milk product that was chemically acidified with ortho-phosphoric acid (placebo). The viable bacteria in the products were enumerated by plating of appropriate dilutions and colony forming unit determination. Intervention products were taken orally, one serving per day (100 mL) during breakfast.

Subjects participated in an approximately 100-day-long trial with 3 intervention periods (Fig. 1). Subjects collected ileal effluent samples after breakfast, for metataxonomic analysis, at home or during test days in the testing facility in 15 mL collection tubes (screw cap faeces container tubes, Sarstedt, Germany) containing a DNA stabilisation solution (2X concentrate DNA/RNA Shield, Zymo Research, CA, USA) on days 1, 7, and 13 during the run-in period, on days 3, 6, 9, and 12 of each intervention period, on days 7 and 13 of each wash-out period and on days 7 and 14 of the run-out period. The collected samples were stored in a portable freezer (− 18 °C ± 4 °C), given to the study subjects prior the start of the trail. To circumvent differences in daily transit time or transit time differences between individuals, we used for most of the analysis the average abundances. Moreover, from a following up study, we know that within the first 12 h post-consumption we do detect the PDB (Zaccaria et, manuscript in preparation). At each test day (V1–V6), a large volume (50–100 mL of ileostomy effluent) for metatranscriptome analysis was collected during the first five hours after taking a standardised breakfast (that included the intervention product), and was directly mixed with RNA-later (Sigma, Germany), stored overnight at 4 °C ± 2 °C, and then transferred to a freezer (− 80 °C ± 4 °C). At the same sampling timepoint, first morning urine and a small effluent sample (2 mL and 15 mL respectively) was collected for urine metabolites and ileostomy effluent SCFA profiling both kind of samples were immediately stored at − 80 °C until analysis, as detailed previously [33] and Supplementary materials and methods. At the start and end of each intervention period gastro-intestinal (GI) permeability was assessed by a mixed sugar test (Additional file 1: Supplementary information).

Compliance to dietary interventions and to the restrictions was checked by asking whether subjects consumed the products and whether they were able to abstain from food on the forbidden food list on every test day, revealing only a few minor deviations (Additional file 1: Supplementary Table 1). Gastro-intestinal symptoms, occurrence, and severity of adverse events during and after consumption of the three products were reported and recorded during the entire study period (Fig. 1).

### Intestinal microbiota composition

Baseclear (Leiden, The Netherlands) performed the DNA extraction from the ileostomy effluents, using DNA Fecal/Soil Microbe Kits (Zymo, CA, USA) according to manufacturer’s instruction and also generated and Illumina-sequenced (MiSeq) the 16S rRNA gene amplicon libraries (V3–V4 region, primer 341F and 805R). The results were analysed using CLC Genomics Workbench version v7.5.1 and the CLC Microbial Genomics Module version 1.5 (CLC bio, Arhus, Denmark). Briefly, the paired end reads were merged into one by CLC Workbench with default setting, and CLC pipeline was used for primer and quality trimming. The remaining high-quality sequences were clustered into operational taxonomic units (OTU) using the SILVA 16S v132 97% database as mapping reference database. A total number of paired-end, unique reads of ~ 14 M, with an average of 36,309 reads/samples, were clustered in 11,939 operational taxonomic units (OTUs) of which 10218 with more than 2 counts. After removal of the outlier samples the reads were clustered in 9270 OTUs. When investigating the interventions’ effect on the endogenous microbial community, the PDB-related OTUs were removed followed by recalculation of the relative abundances of the remaining OTUs

OTUs representing *L. rhamnosus* were investigated in a follow-up study where a subgroup of the ileostomy subjects enrolled in the present study consumed a standardised breakfast with a single dose of *L. rhamnosus* CNCM I-3690 product, identical to those used in the 2-week intervention period. Subsequently, complete ileostomy effluent output has been collected, over time intervals of 4 hours during the first 12 h following consumption of the products. These samples were used to assess the population size and qPCR, as well as the corresponding effects on SI microbiota composition via 16S analysis. The specificity of the primers used (FOR AS113: GTGACAACCGCAATCACTTG, REV AS114: TATCGGTGCCATTGAGTGAA), targeting a gene encoding a putative transcriptional regulator of the Cro/CI family, was verified both in silico via BLAST search paying particular attention to align the sequences with genomes of bacterial genera typically found in ileostomy effluents, and *in vitro* performing PCR and qPCR on DNA extracted from ileostomy effluents sampled prior *L. rhamnosus* product consumption (Supplementary figures 20 and 21).

Correlation analysis between the qPCR estimated copies of its genome and *L. rhamnosus* relative abundance obtained via 16S compositional analysis confirmed that the OTUs assigned by CLC workbench to *L. rhamnosus* accurately represented *L. rhamnosus* CNCM I-3690 (Supplementary Figure S22).

Unfortunately, similar analysis could not be performed for the OTUs assigned to *S. thermophilus* by CLC-workbench due to a lack of discriminating nucleotides within the V3–V4 amplicon sequence relative to (a fraction of) the endogenous small intestinal *S. salivarius* population, which could also not be resolved by DADA2 based ASV analysis [34] (data not shown). However, strain-specific qPCR targeting the CRISPR region of *S. thermophilus* CNCM I-1630 established that the ileostoma effluent excretion curves of *L. rhamnosus* CNCM I-3690 and *S. thermophilus* CNCM I-1630 in the first 12 h post-consumption of the respective products was very comparable (Supplementary Figure S23), which is also in agreement with the observation that the volunteer specific average relative abundances assigned by CLC workbench to *S. thermophilus* and *L. rhamnosus* was clearly correlated (Supplementary Figure S24). The specificity of the primers used for S. *thermophilus* CNCM I-1630 (FOR OFF2540: CTATCGAACATTTACGAGCTG, REV OFF2541: GTATCTGTTGAAAGAGGTGTG), targeting the CRISPR region of *S. thermophilus* CNCM I-1630, was done similarly with the ones of *L. rhamnosus* CNCM I-3690. They were verified both in silico via BLAST search and *in vitro* performing PCR and qPCR on DNA extracted from ileostomy effluents sampled prior Yogurt (fermented by *S. thermophilus* CNCM I-1630 and *L. bulgaricus* CNCM I-1519) consumption (Supplementary Figure S20 and S21). These findings suggest that the *S. thermophilus* relative abundance levels detected by CLC workbench represent an appropriate approximation for the *S. thermophilus* CNCM I-1630 population per sample. In conclusion, the metataxonomic detection and quantification of *L. rhamnosus* CNCM I-3690 is highly reliable, and although less precise we considered the metataxonomic quantification of *S. thermophilus* obtained by CLC workbench sufficiently reliable for further analysis and used the resulting *S. thermophilus* relative abundances per sample throughout the rest of this manuscript as the best-possible approximation of this species’ (i.e., PDB) population size.

### Metatranscriptomics

Ileostomy effluent samples collected for this analysis (see above) were thawed and homogenised while remaining cooled on ice using Ultra-Turrax t50 (IKA, Germany). Ten millilitres of the homogenised samples was transferred to a tube and larger debris was removed by low-speed centrifugation (3’, 500×*g*, 4 °C, with the centrifuge’s breaks switched off). Subsequently, bacteria and smaller-sized debris were pelleted by higher speed centrifugation (10’, 8000×*g*, 4 °C) and the pellet obtained was immediately resuspended in 100 μl of phenol–chloroform–isoamyl alcohol (pH 6.5–8.0, Sigma, Germany) followed by RNA extraction using the RNeasy PowerMicrobiome Kit (Qiagen, Germany) according to the manufacturer’s instructions. Extracted RNA was stored at – 80 °C. RNA quality and quantity were analysed by agarose gel electrophoresis as well as using the TapeStation 2200 (Agilent Technologies, CA, USA). Obtaining RNA of sufficient quality in these samples was challenging, resulting in a restricted and unbalanced sample size for this analysis. Most of the successfully sequenced samples were taken at the start or end of the fermented product intervention periods and only for three subjects a complete sample set (all 6 samples) could be analysed. The 250~300 bp insert cDNA library with rRNA removal (Ribo-ZeroTM Magnetic Kit, Illumina, USA) and sequencing were performed by Novogene (Hong Kong) using the HiSeq2500 platform (PE150, 12 G raw data/sample). HUMAnN2, with the default settings [35], was used for the functional profiling of the metatranscriptome datasets by mapping against UniRef90 protein database (updated global profiling of the Human Microbiome Project [36]) and MetaCyc database 19.1 [37] to obtain the bacterial pathway abundances (Functional Metatranscriptome Mapping) combined with taxonomic profiling by the included taxonomic identification tool *MetaPhlAn2*.

### Data mining and statistics

Descriptive statistics were calculated for age, BMI and gender (Supplementary Table S2). SCFA and intestinal permeability data was analysed using IBM SPSS statistics 25 (IBM Corporation, Armonk, NY, USA) and a Kolmogorov-Smirnov tests as well as a visual check of normality (QQ plot) of the data were performed.

Intervention effects on SCFA and GI permeability were assessed by mixed model analysis on baseline-corrected data. A *p* < .05 was considered statistically significant.

Following removal of the biological outlier (Kock’s pouch subject; see “Results” section) the microbiota compositional data was filtered for at least 2 reads in 10% of the samples, MicrobiomeAnalyst [38] was used for the calculation of α-diversity indices (observed species, Shannon, and Chao1). Microbial compositional data as well as the Functional Metatranscriptome Mapping β-diversity, distance-based redundancy analysis (db-RDA), principal component analyses (PCA), partial, and non-redundancy analysis (RDA), were assessed using the Canoco 5.10 software suite [39] using 1000 permutations to assess significance and, where needed relative abundance values were log transformed (Y’ = Y + 1000) and centred. When required, and prior to statistical analysis in GraphPad Prism (8.3.1 for Windows, CA, USA), the data were initially tested for normal distribution using the D’Agostino and Pearson omnibus normality algorithms and, subsequently analysed with either analysis of variance (ANOVA) and Tukey’s Post-test, or Kruskal-Wallis test and Dunn’s Multiple Comparison Post-test, for normal and non-normal distributed data, respectively. A *p value*, or, when needed, a *corrected p value* < .05 was considered statistically significant. The parameters utilised for the core microbiome analysis were set at a sample prevalence ≥ 30% and a relative abundance ≥ .01%.

Multivariate association with linear models (MaAsLin2) analysis with default setting was used for the association of PDB with bacterial pathways. Differential expression and abundance analysis were performed by Empirical Analysis of Digital Gene Expression (EdgeR) [40] implemented in Network Analyst [41] using subject as secondary, block factor. For both MaAsLin2 and EdgeR a false discovery rate (FDR) was used to correct for multiple testing and FDR adjusted *p* < .05 was considered statistically significant.

## Results

### Study population, compliance, adverse events, and secondary outcomes

For this study, 18 ileostomy subjects were screened of which 16 (Fig. 1, Additional file 4: Supplementary CONSORT flow diagram) were enrolled. Fifteen subjects completed the protocol (see Materials and Methods). In general, compliance was high and no serious adverse events were reported during this trial (Additional file 1: Supplementary Table S1, Supplementary Figure S2).

The primary aim was to determine the impact of the consumption of fermented dairy products on the small intestine microbiota composition and activity. In addition, intestinal permeability and SCFA levels in ileostoma effluent samples were determined at the start and end of each of the intervention periods. Notably, neither permeability nor SCFA amount or composition was significantly affected by any of the interventions (Additional file 1: Supplementary Figure S3, S4, and S5).

### Longitudinal metataxonomic analysis of the small intestinal microbiota

Longitudinal microbial composition in ileostomy effluent was analysed by metataxonomic analysis, which was successful in 390 (> 90%) of the 432 collected effluent samples (27 per individual). The samples obtained from two subjects had the lowest success rate, which for at least one of the subjects appeared to be due to the low DNA recovery in the samples (Additional file 1: Supplementary Table 3). There was no difference in the success rate in obtaining data across the different periods of the trial (Additional file 1: Supplementary Table S4). Initial principal component analysis (PCA) analysis of the microbiota data obtained (Additional file 1: Supplementary Figure S6A) revealed that all samples obtained from one subject strongly deviated from the rest. This subject was the only individual with a Kock’s pouch rather than the standard ileostoma, which led us to exclude this subject in further analyses as a biological outlier. In the remaining samples, 9270 operational taxonomic units (OTUs) were identified, representing 258 genera, and 9 phyla.

The majority of the sequences were assigned to Firmicutes (78.6%) and Proteobacteria (11.9%), followed by Actinobacteria (6.3%) and Bacteroidetes (3.1%). The average microbiota composition per subject revealed a high degree of variation among the subjects (Supplementary Figure S6C, D). Redundancy analysis revealed that almost half (46.6%) of the overall microbiota composition at species level was explained by inter-subject variation (Additional file 1: Supplementary Figure S6B). In addition, longitudinal composition analysis per subject revealed remarkable time-dependent fluctuation during the study period, although composition appeared more stable in some individuals (Additional file 1: Supplementary Figure S6E and F). Nevertheless, intra- and inter-subject comparison of Bray-Curtis dissimilarity during the study showed a higher degree of variance between subjects than within subject (Additional file 1: Supplementary Figure S7). These metataxonomic analyses expand insights gained in previous studies [14, 15], by confirming the high degree of variance of the SI microbiota composition between and within volunteers, while establishing that a personal microbiota signature is recognisable despite temporal fluctuation.

### Impact of fermented dairy product consumption on the SI microbiota composition

The fermented milk products consumed during the study contained either *Lacticaseibacillus rhamnosus* CNCM I-3690 or the yogurt starter culture bacteria *Streptococcus thermophilus* CNCM I-1630 and *Lactobacillus delbrueckii* subsp*. bulgaricus* CNCM I-1519. The placebo control product was unfermented, acidified milk. In agreement with the relatively low numbers of *L. bulgaricus* (see Materials and Methods) in the yogurt product, this species remained undetected in the effluent microbiota following consumption. Thereby, detection of product-derived bacteria (PDB) in the effluent microbiota was restricted to the abundant bacteria in the respective products, i.e. *L. rhamnosus*, and *S. thermophilus*. Importantly, *L. rhamnosus* was not detected in any sample obtained outside of the product-specific intervention periods, showing that this organism transiently inhabits the small intestinal niche and rapidly disappear once consumption is stopped and establishing that the 2-week washout suffices to avoid microbial carry-over between intervention periods, which is also confirmed by the absence of a detectable difference between the last samples obtained during the wash-out period and those obtained during the run-in (Additional file 1: Supplementary Figure S8). Moreover, strain specific quantification of *L. rhamnosus* CNCM I-3690 by quantitative PCR corresponded very well with the metataxonomic derived relative abundance (see methods for more details and supplemental Figure S22). Notably, the metataxonomic detection of *S. thermophilus* CNCM I-1630 was not completely reliable due to the presence of endogenous *S. salivarius*. Nevertheless, several confirming experimental observations, including strain-specific qPCR analyses, supported that the metataxonomic analysis performed was the best possible approximation of the relative abundance of this product derived bacterial species (PDB), and led us to employ the OTUs assigned to *S. thermophilus* as a proxy for the abundance of *S. thermophilus* CNCM I-1630 (i.e. PDB for the yoghurt intervention) per sample (see methods for details and supplementary Figure S23 and S24) throughout the rest of this study.

**Fig. 2:**
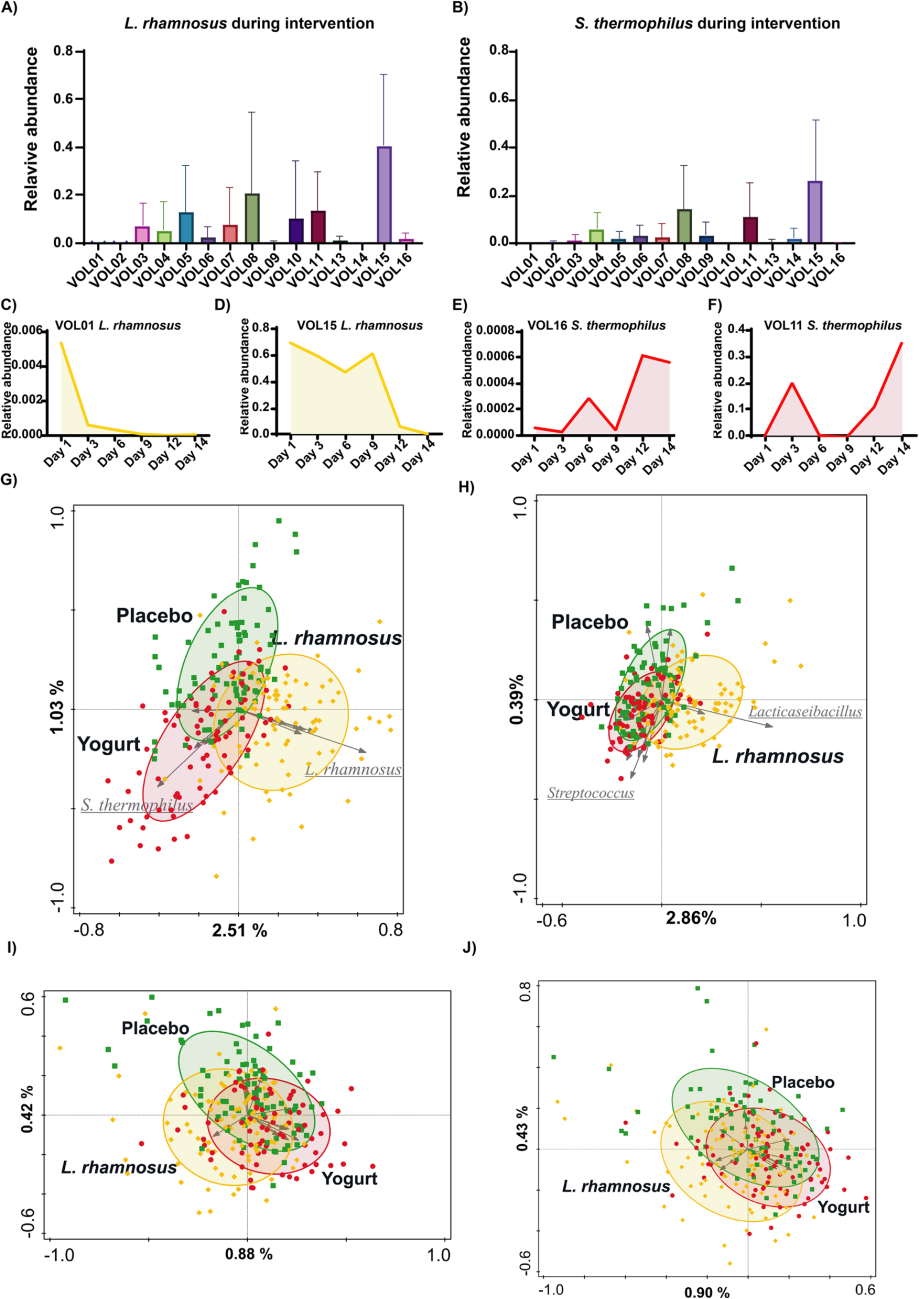
Microbiota impact of the consumption of fermented products. PDB could be detected in the samples obtained during the corresponding intervention period. *L. rhamnosus* and *S. thermophilus* constituted 8.5%, and 5.2% of the total microbiota on average, but displayed extreme variation in intra- and inter-individual comparison (**A**–**F**). Subject corrected RDA analysis separated samples obtained during the different intervention periods at species (**G**, *p *= 0.001, explained variation 2.7%) and genus (**H**, *p* = 0.001, explained variation 2.03%) level. Samples are coloured by intervention period (yogurt [red], placebo [green] and *L. rhamnosus* [yellow]), and the microbial species or genera with the strongest contribution to the separation are indicated, with the PDB underlined. Subject corrected RDA analysis of the microbial composition of the intervention period samples after removal of the OTUs corresponding to PDB indicated that samples could still be significantly separated on basis of the intervention period in which they were taken, albeit with much less variation explained (species level, **I**; genus level, **J**; explained variation 0.44% and 0.48%, *p* = 0.024 and 0.036 respectively)

In samples obtained during the intervention periods, PDB corresponding to the intervention product were detected while they were consistently absent in samples collected during the rest of the study (Fig. 2A–D). On average, *L. rhamnosus* and *S. thermophilus* constituted 8.5%, and 5.2% of the total microbiota in the effluent samples collected during the 2 intervention weeks. However, highly variable relative abundances were observed when comparing intra- and inter-personal samples. Illustratively, 3 subjects had a PDB average relative abundance below 1% (max 5%) and the PDB could not be detected (< 5.7E−03) in some of the samples, whereas 3 other subjects had a PDB average abundance above 10%, including the striking maximum relative abundance of *L. rhamnosus* at 88.2% in one of the samples. These observations exemplify the strong oscillation of relative abundance of the PDB in all subjects (Fig. 2A–D), supporting the dynamic nature of the small intestinal microbial community.

Despite the large impact of the PDB in some samples, the alpha diversity of the effluent microbiota remained unchanged during the intervention periods as compared to the run-in, although alpha diversity varied substantially between the subjects (Additional file 1: Supplementary Figure S9). The impact of the intervention on the microbiota composition was analysed by subject corrected redundancy analysis (RDA), revealing a significant effect of the intervention at both genus and species level, albeit that these effects only explained a small fraction of the overall variation (Fig. 2H, G). The drivers of the significant separation of the intervention-specific samples were the PDB (Fig. 2G). Moreover, species-level beta diversity analysis revealed that the samples obtained during the interventions were significantly different (Additional file 1: Supplementary Figure S10), which was supported by the inclusion of the PDB in the core microbiome of the effluent samples during the respective intervention periods. Notably, the PDB addition to the core microbiome was the sole difference detected in this analysis (Additional file 1: Supplementary Figure S11).

### Interventions’ negligible effects on the SI endogenous microbiome

The predominant effect of the product interventions on the small intestine microbiota composition appears to be the presence of PDB in samples collected during the intervention period. To investigate the possible effect of the interventions on the endogenous microbial community, the PDB-related OTUs were removed followed by recalculation of the relative abundances of the remaining OTUs. The resulting dataset was used for subject corrected RDA analysis demonstrating that samples could still be significantly separated on basis of the intervention period in which they were taken (Fig. 2I, J), albeit that samples displayed a large overlap, and only a very low amount of variation could be explained (0.44% and 0.48% at species and genus level, respectively). The 11 endogenous species associated with the different interventions were detected by empirical analysis of digital gene expression (EdgeR) differential abundance analysis, correcting for subject ID (Additional file 1: Supplementary Table S5). However, none of these species were prevalent among the majority of subjects or present at relative high abundance (max prevalence 27%, max abundance 1.5%). These findings indicate that the intervention-impact on the endogenous microbiota is negligible as poorly conserved among subjects, which agrees with the minimal amount of explained variation found by RDA and suggests an absence of a meaningful effect.

### Endogenous Peptostreptococcaceae abundance is correlated with the relative abundance of PDB during intervention

**Fig. 3:**
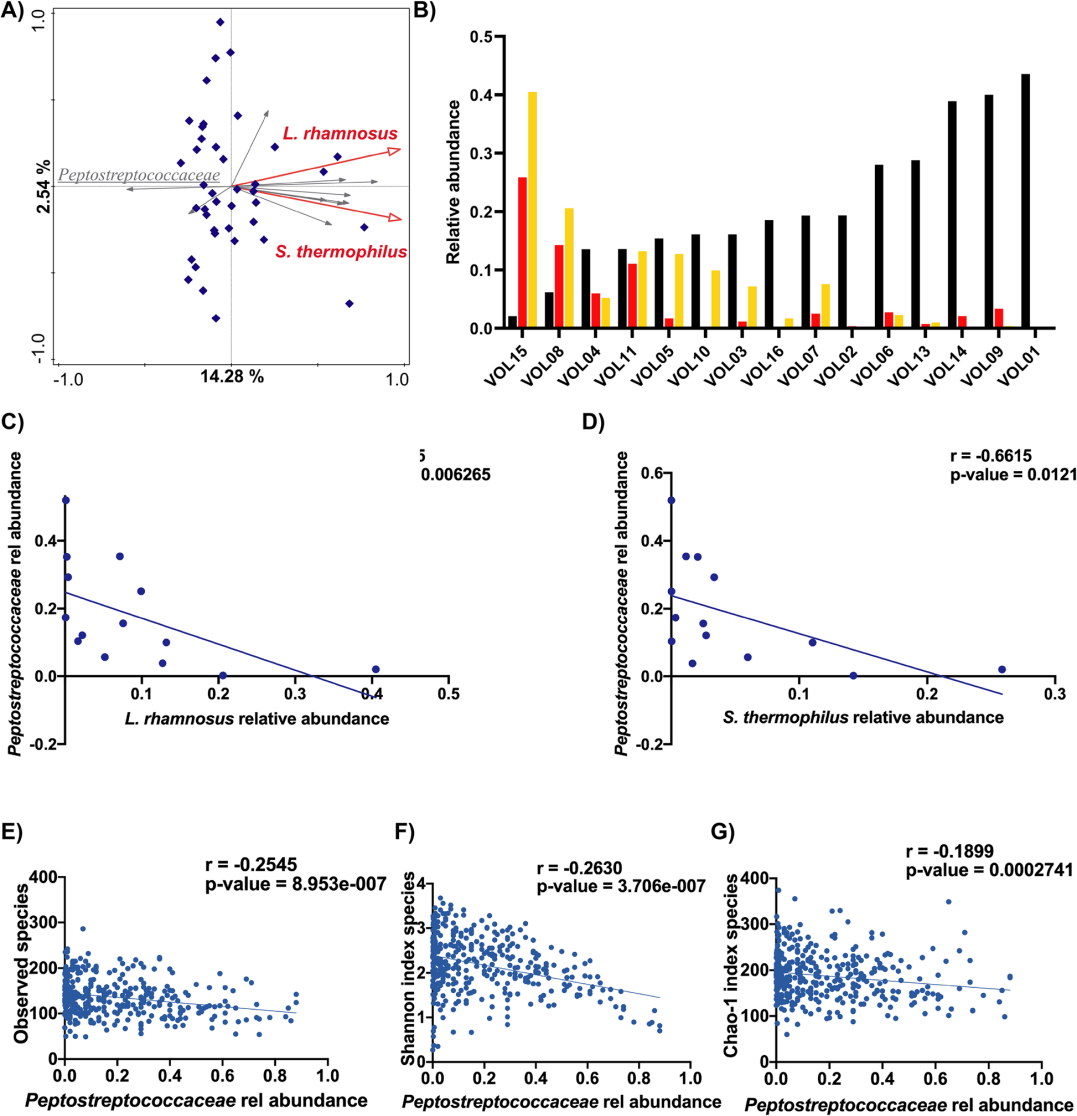
Association of Peptostreptococcaceae abundance with PDB and alpha diversity. **A** RDA analysis of the microbial composition of the run-in samples shows a negative association of participant-specific average relative abundances of *L. rhamnosus* and *S. thermophilus* during the intervention periods (indicated by the red arrows) with the Peptostreptococcaceae family relative abundance (explained variation 12.33%, *p* = 0.002). **B** Per individual average relative abundance of *L. rhamnosus* (yellow), *S. thermophilus* (red), and *Peptostreptococcaceae* (black) in samples taken during the respective intervention periods, and the run-in period, respectively. **C**, **D** Spearman correlation of average relative abundance of Peptostreptococcaeae in the run-in samples and average relative abundance of *L. rhamnosus* (**C**) and *S. thermophilus* (**D**) in the respective intervention period samples. Panel E&F: Relative abundance of the Peptostreptococcaceae family in all samples is negatively correlated (Spearman) with microbiota alpha diversity, reflected by richness (**E, G**) and evenness (**F**)

The analyses above indicated that the average relative abundance of the PDB during the intervention periods differed substantially per subject (Fig. 2A, B). Remarkably, high congruency was observed between the subject-specific average relative abundance of *S. thermophilus* and *L. rhamnosus* during the respective interventions, suggesting PDB colonisation efficiency is very subject specific but independent of the PDB species. This led us to investigate whether the endogenous baseline microbiota could explain these differences in abundance of *L. rhamnosus* and *S. thermophilus*. The corresponding RDA analysis revealed that while several bacterial families appeared to be enriched in subjects that displayed high relative abundance values for the PDB, only the *Peptostreptococcaceae* were negatively associated with the detected abundance of the PDB (Fig. 3A). The significant negative correlation between PDB colonisation efficiency and endogenous *Peptostreptococcaceae* relative abundance was confirmed by Spearman correlation analysis (Fig. 3C, D). Analysis of the genera-composition of the *Peptostreptococcaceae* family revealed an overall relatively simple community at genus level that displays a high degree of compositional fluctuations over time within individuals and appears commonly to be domination by unclassified / uncultured family members besides *Romboutsia*, with occasional blooms of *Peptostreptococcus*, *Terrisporobacter*, and *Paeniclostridium* (Supplementary Figure S18 and S19). Due to the not conserved composition of the *Peptostreptococcaceae* family among and within subjects, no significant correlation could be observed between any *Peptostreptococcaceae* genera and PDB.

Notably, despite substantial variation within and between subjects, we could not detect a correlation between the microbial concentration of the ileostoma effluent samples and *Peptostreptococcaceae* or PDB average relative abundance (Supplementary Figure S12C, D), suggesting that the abundances of neither *Peptostreptococcaceae* nor PDB is associated with a difference in bacterial density. The microbial density data were collected in an additional experiment where samples were collected during the first 12 h after consumption of *the L. rhamnosus* fermented product in the morning, and in these samples, the amount of *L. rhamnosus* via qPCR as well as the 16S metataxonomic composition were determined. Using the qPCR data of *L. rhamnosus* in combination with its relative abundance in the sample, we were able to estimate the community density in these samples, which did not correlate with *L. rhamnosus* relative abundance nor with the *Peptostreptococcaceae* relative abundance, showing that the relative abundance observed is not explained by microbiota density variation (Supplementary Figure S12C and D). Intriguingly, high *Peptostreptococcaceae* relative abundance was correlated with a lower microbiota diversity (Fig. 3E–G).

### Colonisation efficiency of *L. rhamnosus* is associated with elevated expression of carbon fermentation pathways in the small intestine microbiota compared to the yogurt intervention

The microbial activity in ileostomy effluent was analysed by metatranscriptome analysis in the samples obtained on the first and last days of the intervention periods. Functional metatranscriptome mapping (FMM) of the small intestine microbiome were obtained for each sample by genome, protein and pathway mapping. To understand how well the microbial composition identified by 16S rRNA reflected the taxonomic distribution within the metatranscriptome, a detrended correspondence analysis (DCA plot) was used and it revealed a substantial overlap between the compositional profiles (Supplementary Figure S1).

Inter subject FMM differences was the predominant source of variation, explaining almost 25% of the overall FMM variance (Additional file 1: Supplementary Figure S13A). This is supported by the higher Bray-Curtis distance between FMM data obtained for different subjects, compared to longitudinal FMM data from a single individual irrespective of the intervention period (Additional file 1: Supplementary Figure S13B). Nevertheless, subject-corrected RDA analysis revealed that the interventions did significantly affect the FMM and could explain 4.64% of the overall variance in the FMM data (Additional file 1: Supplementary Figure S14). The differentially expressed microbial pathways that underlie the intervention-associated effect on the FMM were identified using EdgeR differential expression analysis, with subject ID as a co-variate. This revealed that only expression of the L-rhamnose degradation I pathway was significantly increased in the FMM associated with the *L. rhamnosus* intervention relative to the placebo intervention (Additional file 1: Supplementary Table 6), while no FMM effects were found when comparing yogurt and placebo interventions. Notably, comparative FMM analysis of samples obtained during the yogurt and *L. rhamnosus* interventions revealed the increased expression of 8 carbon- and fermentation- associated pathways during the *L. rhamnosus* intervention, including the *L-rhamnose* degradation I pathway (Additional file 1: Supplementary Table 6). These results show that the consumption of the *L. rhamnosus* fermented product associates with elevated expression of carbon metabolism pathways in the small intestinal microbiota, particularly when compared to the consumption of the more traditional yogurt. This elevated expression is especially apparent for the L-rhamnose degradation pathway.

### Endogenous microbiome pathway activity patterns influence the colonisation efficiency of PDB

**Fig. 4:**
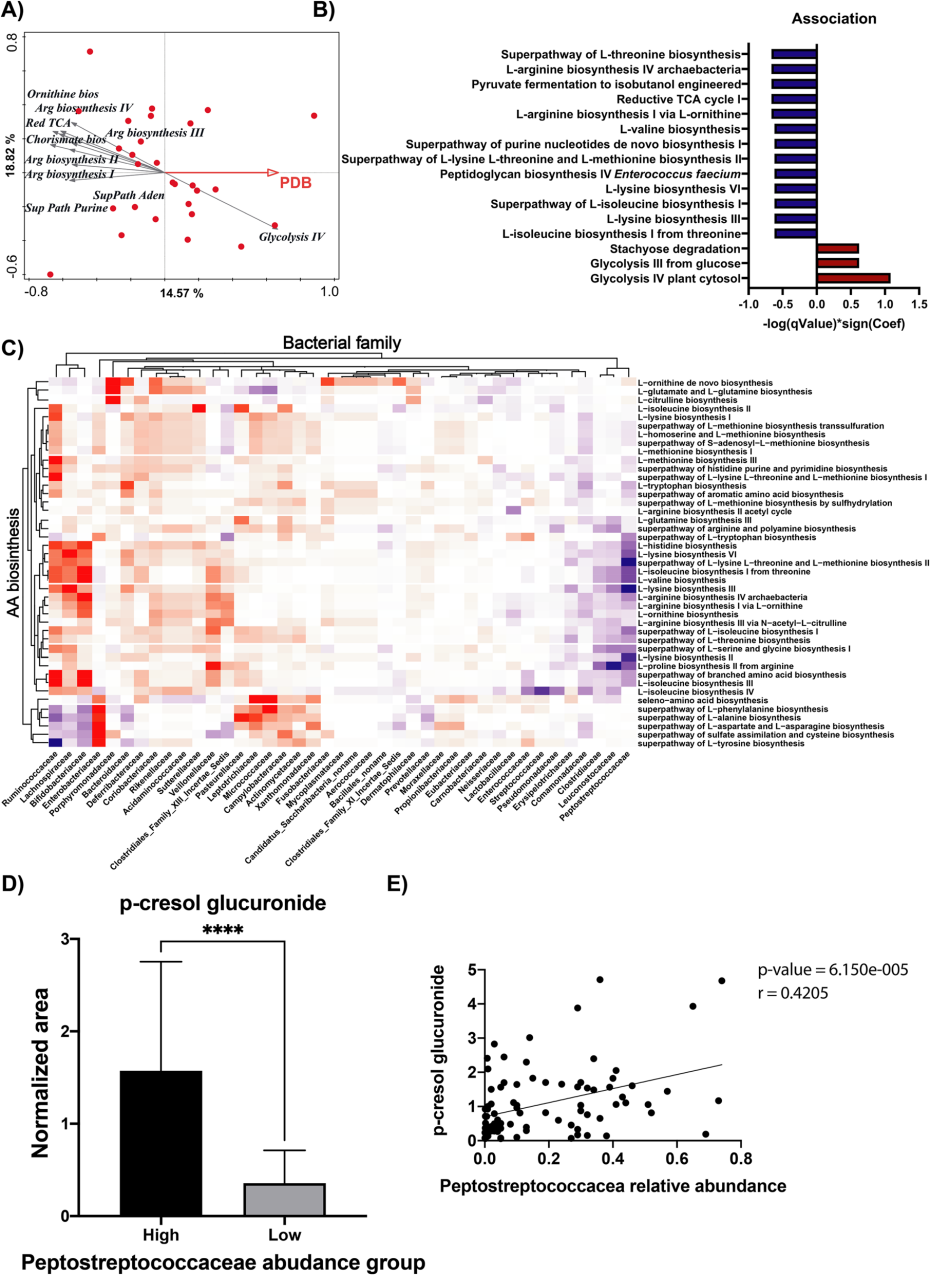
Small intestinal microbiota activity associates with the temporary colonisation by PDB. **A** RDA using FMM determined during the intervention periods involving fermented products (*L. rhamnosus* and yogurt) is associated with average relative abundance of PDB during the same intervention period (red arrow), displaying top 10 strongest associated pathways (grey arrows) (explained variation: 9.83%, *p* = 0.004.). **B** MaAsLin2 analysis confirmed both positive, and negative associations of PDB average abundance with microbiome activity levels of the glycolytic pathway (and expanded this observation to the Stachyose degradation pathway), and various amino acid metabolism pathways, respectively. **C** Amino acid biosynthesis pathway activity associates with a variety of bacterial families (Pearson correlation), with the notable exception of the Peptostreptococcaceae that is among the three families that negatively correlate with amino acid biosynthesis activity in the FMM. **D**, **E** p-creosol glucuronide, microbial metabolites generated through proteolytic fermentation and found in urine, positively correlates with Peptostreptococcaceae relative abundance (**E**, Spearman rank) and it is found in significant higher amount in subjects with a high Peptostreptococcaceae abundance (high: Peptostreptococcaceae relative abundance > 0.15, Mann-Whitney test, *p* < .0001)

To investigate whether variations in the FMM data were associated with the highly variable and subject-specific relative abundance of PDB during the intervention periods, RDA analysis was performed using the FMM determined during the intervention periods and, as explanatory variable, the average relative abundance of PDB during their respective interventions (Fig. 3A). A strikingly strong enrichment was found for various bacterial pathways related to amino acid metabolism in samples displaying the lower PDB relative abundance, which was contrasted by an association of glycolytic pathway expression (i.e., Glycolysis IV in EcoCyc) with the higher PDB relative abundance (Fig. 3A). These findings were confirmed using the multivariate association with linear models (MaAsLin2) analysis, which also pointed to positive associations of PDB abundance and glycolytic pathway activity (Glycolysis IV and Glycolysis III), while expanding this to the Stachyose degradation pathway (Fig. 3B). MaAsLin2 also confirmed the negative association between PDB abundance and the expression of the majority (9 out of 12) of amino acid biosynthesis pathways identified in FMM (Fig. 3B). Subsequently, metagenomic phylogenetic analysis (MetaPhlAn) of the FMM data enabled the phylogenetic classification of these differentially expressed pathways, revealing that the activated amino acid metabolism pathways were assigned to a broad range of bacterial families in a scattered manner (Fig. 3C), encompassing most of the microbial families encountered within the small intestinal ecosystem (30 out of 41). In this context, it is striking that the family of the *Peptostreptococcaceae* is one of the few bacterial taxa that negatively correlated with the amino acid metabolism pathways in the FMM. To further support the link between *Peptostreptococcaceae* and acid-derived energy metabolism, we analysed urine microbial metabolites generated through proteolytic fermentation both via correlation analysis (spearman rank, Fig. 4E, and Supplementary Figure S15) and by dividing the subjects into two groups defined by the average abundance of *Peptostreptococcaceae* (high: *Peptostreptococcaceae* relative abundance > 0.15). Most of the metabolites analysed positively correlated with the relative abundance of the *Peptostreptococcaceae* family, which was observed both in a comparative analysis of individuals that had a high and low abundance of this bacterial family, but also when individual measurements were used, albeit both analyses clearly also demonstrated that this relationship is somewhat noisy. Cresol is a methyl phenol produced via microbial degradation of tyrosine [42], phenylacetylglutamine as well as 4-hydroxyphenylacetylglutamine result from glutamine conjugation of phenylacetic acid, which is almost exclusively derived from the microbial conversion of phenylalanine [43] (Fig. 4D, E and Supplementary Figure S15). The results confirmed the enhanced proteolytic fermentation in case of high abundance of *Peptostreptococcaceae*, reflected by higher level of microbial metabolites generated through proteolytic fermentation urine.

Taken together, our results highlight how the endogenous microbiota composition and its predominantly active energy metabolism could explain the individual-specific PDB colonisation efficiency, specifically identifying the *Peptostreptococcaceae* family as a microbial group that prominently reflects, and is likely involved in, these individualised effects.

## Discussion

In this study, we present the longitudinal metataxonomic study of the small intestine microbiota, including diet intervention periods to assess the microbial impact of the consumption of bacteria-rich fermented dairy products. Although metataxonomic analysis was the focus of this study, we also determined whether the interventions affected small intestinal mucosal permeability or the SCFA composition of the stoma effluent, revealing that neither of these parameters was significantly affected during the dairy product interventions. Nevertheless, the SCFA measurements confirmed previous observations that substantial amounts of microbial fermentation end-products are already formed in the small intestine [14], suggesting that their profound effects on mucosal and systemic functions of the host [44] is exerted throughout the length of the intestinal tract rather than predominantly in the large intestine. The permeability measurements in this study were inspired by previous studies showing that *L. rhamnosus* CNCM-I 3690 (the strain that was also used here) could restore impaired intestinal barrier functions in mice [27, 28]. The lack of confirmatory observations in the present cohort of ileostomists may be due to the fact that contrary to the studies using mouse models, the ileostomists participating in this study did not suffer from impaired mucosal barrier function, minimising the potential of dietary interventions to elicit an improvement, illustrative of translational difficulties across different mammalian models [45].

Our metataxonomic results confirmed the previously reported high degree of difference of the small intestinal microbial composition between and within subjects [15, 46]. In the present study almost half (46.6%) of the overall gut microbiota variation was determined by the individual. Although extensive longitudinal fluctuations within an individual were detected, a personal microbiota composition signature was recognisable, analogous to what has been found in the faecal microbiome [47, 48]. Metataxonomic analyses also revealed striking variation in the average relative abundance of the PDB between and within individuals. Notably, whereas the relative abundance determinations of *L rhamnosus* CNCM-I 3690 could be shown to be highly reliable and accurate, such strong confirmation could not be obtained for the relative abundances of *S. thermophilus* CNCM I-1630. However, the OTUs assigned to *S. thermophilus* appeared to adequately provide an approximation of the relative abundance of this PDB and were therefore used as a proxy for the population size of the PDB *S. thermophilus* CNCM I-1630 during the yoghurt intervention period. Despite the time- and individual-associated microbiota composition variability the PDB were part of the core microbiome during the respective interventions in all subjects. Moreover, the PDB were the predominant determinants in the metataxonomic separation between the intervention periods and the run-in and intermittent wash-out periods, demonstrating how consumption of the fermented intervention products may impact the SI microbiota in a much higher degree when compared to the bacterial estimate usually found in faecal samples [49, 50]. In fact, in some subjects the PDB comprised a fluctuating but large proportion of the overall small intestine microbiome, reaching up to 88% relative abundance, whereas in other subjects the PDB relative abundance was consistently low in all samples collected. These results are in agreement with an invasive endoscope perfusion study in healthy subjects that showed that *Lactocaseibacillus casei* transiently amounted up to 75% of the ileal microbiota community in only one of the four participating individuals [20]. Similarly, highly individualised colonisation patterns have been reported for bacteria derived from fermented food products and probiotics in studies focussing on the faecal microbiota [51]. This led to the hypothesis that colonisation-permissive and colonisation-resistant endogenous microbiome communities determine this individual-specific colonisation efficacy [21]. However, the high impact of food-borne bacteria is uniquely observed in the small intestine, since studies targeting the fecal microbiome described much more subtle effects in terms of the transient PDB relative abundance [20, 49]. It is of importance to notice that ingestion of the fermented products had a negligible impact on the endogenous microbiota, possibly also due to the high degree of difference of the SI microbiota between and within subjects.

We expanded the metataxonomic analysis in this study with metatranscriptome analysis of the small intestine microbiota at the start and end of each of the intervention periods, using genome mapping and FMM analysis to determine the activity profile of the small intestine microbiome. Notably, comparative analysis of the metatranscriptome FMM patterns also showed that the individual from which the sample was taken was the co-variate that explained most of the observed variance in FMM (~ 25%). Nevertheless, the intervention period could explain approximately 5% of the total FMM variance, identifying the activation of the *L-rhamnose* degradation pathway during the consumption of the *L. rhamnosus* fermented product relative to the other interventions. This may be related to presence of this pathway in the *L. rhamnosus* genetic repertoire (data not shown) and its low expression level by the endogenous microbiota. This analysis illustrates that metatranscriptome analyses reveals a comprehensive and individualised functional view of the microbiome and allows to detect the impacts of diet intervention and/or transient PDB abundance at functional level.

The striking variation in subject-specific relative abundance of the PDB allowed a stratified interpretation of the metataxonomic data, identifying the strong association of the abundance of the *Peptostreptococcaceae* family in the endogenous microbiota and the capacity of the PDB to effectively occupy this ecosystem in a transient yet robust manner. High *Peptostreptococcaceae* abundance correlates with lower microbial diversity of the endogenous microbiota and a lower abundance of PDB, expanding the previously proposed probiotic-permissive and probiotic-resistant faecal microbiota hypothesis [21] to the small intestine and pinpointing *Peptostreptococcaceae* as an indicator of a resistant SI microbial community. Members of this family may generically limit the inhabitation of the niche by other microbial groups, resulting in lower diversity, but also suppressing transient colonisation by PDB. Metatranscriptome FMM analysis demonstrated that higher relative abundance of the PDB is associated with a microbial ecosystem that executes a carbon-derived energy metabolism, whereas lower PDB abundance associates with a variety of amino acid metabolism pathways that are assigned to a variety of microbial families within the ecosystem, notably excluding the *Peptostreptococcaceae*. These results imply that an environment rich in carbohydrates creates a favourable niche for the temporal colonisation of by both *L. rhamnosus* and *S. thermophilus*, which agrees with the notion that these bacteria derive most of their metabolic energy from (simple sugar) carbohydrate fermentation [52] In contrast, an environment poor in fermentable carbohydrate sources would direct the endogenous microbiota towards amino acid derived energy metabolism, which could lead to low availability of amino acids that elicits the activation of amino acid metabolism pathways in various microbes. The latter niche characteristics would restrain the colonisation capacity of the PDB, which is supported by the negative association of arginine biosynthesis pathway-activity and the abundance of the arginine auxotrophic *L. rhamnosus* (data not shown). Interestingly, amino acid exchange has been identified as a crucial driver of microbial community interaction, and amino acid cross-feeding is highly prevalent in cooperative communities [53–55]. This agrees with the *Peptostreptococcaceae* as indicators for an amino acid fermenting microbial community, that may be probiotic-resistant, particularly towards the typical saccharolytic probiotics like those belonging to the *Lactobacilaceae* and *Bifidobacteriaceae* families. The ingested bacteria are encountered in protein rich environments where amino acids serve as the predominant energy source, which can include human and animal intestinal samples [56–58]. The analysis of urine metabolites generated through proteolytic fermentation further supports the link between *Peptostreptococcaceae* and amino acid-derived energy metabolism. Moreover, several *Peptostreptococcaceae* are known to ferment (sulphur-containing) amino acids [59], or have been reported to be non-saccharolytic, supporting the importance of amino acid derived energy metabolism in this family [56]. Finally, it has been reported that patients with colorectal cancer had a fecal microbiota enriched with *Peptostreptococcaceae* [60, 61]. This enrichment tended to be reduced after probiotic consumption, underlining a possible interaction between members of this bacterial family and ingested bacteria [60].

## Conclusions

This study shows that the consumption of milk products fermented by *L. rhamnosus* CNCM I-3690, or the yogurt symbionts *Streptococcus thermophilus* CNCM I-1630 and *Lactobacillus delbrueckii* subsp*. bulgaricus* CNCM I-1519 results in both compositional and functional changes of the SI microbiota. These changes are highly personalised, which we propose to correspond to subject-specific composition of the endogenous SI microbiota and its predominating carbon- versus amino acid-derived energy metabolism activity. Moreover, we pinpoint the relative abundance of the poorly characterised bacterial family of the *Peptostreptococcaceae* as an indicator for these subject-specific small intestine characteristics. At present, we do not know the mechanistic foundation of these subject specific small intestinal microbiome characteristics, which could be caused by differences in diet- or behavior-related habits. Alternatively, and analogous to what has been shown for the large intestine microbiome [62, 63] by subject-specific variations in (macro-)physiology of the intestinal tract, like variations in stomach pH, stomach emptying rate, bile and digestive enzyme production levels, or small intestine transit time. Very little is known about these parameters and there is a lack of non-invasive methodologies to accurately determine them in an individual. These potentially important variables are not considered in most diet and microbiota investigations, warranting further investigation of their variation among individuals since they could profoundly affect the relationships between diet, microbiota, and human physiology and health. Thereby these parameters could be critical determinants in individualised metabolic responses to dietary ingredients [64, 65], and may provide a plausible mechanistic explanation for the relationship of the endogenous SI microbiome with the transient colonisation capacity of PDB.

## Supplementary Information

**Additional file 1: Supplementary Methods.** Recruitment criteria, Gastrointestinal permeability, Short Chain Fatty Acids, Intestinal Microbiota composition, Metatranscriptomics, Urine metabolites profiling, Ileostomy effluent bacterial density calculation, Data mining and statistics. **Supplementary Results.** Gastrointestinal permeability and SCFA. **Supplementary Figure 1.** Microbial composition profiles comparison. **Supplementary Figure S2.** Type and severity of gastrointestinal symptoms. **Supplementary Figure S3.** Short chain fatty acid concentration in ileostomy effluents. **Supplementary Figure S4.** Gastro-intestinal permeability. **Supplementary Figure S5.** Short-term gastro-intestinal permeability. **Supplementary Figure S6.** Overall and longitudinal microbiota composition analysis. **Supplementary Figure S7.** Beta diversity of ileostomy effluent microbiota, intra and inter subject; **Supplementary Figure S8.** Carry-over effect analysis. **Supplementary Figure S9.** Alpha diversity of ileostomy effluent microbiota. **Supplementary Figure S10.** Beta diversity of the ileostomy effluent microbiota. **Supplementary Figure S11.** Core-microbiome. **Supplementary Figure S12. ***L. rhamnosus* relative abundance and microbial density in ileostomy samples. **Figure S13.** Inter subject differences functional metatranscriptome mapping analysis. **Supplementary Figure S14.** Intervention products effects on the functional metatranscriptome mapping of the ileostomy effluent. **Supplementary Figure S15.** Analysis of the relashionship between *Peptostreptococcaceae* and urine microbial metabolites generated through bacterial proteolytic fermentation. **Supplementary Figure S18.** Taxonomic composition at genera level of the *Peptostreptococcaceae* family per volunteer. **Supplementary Figure 20.** Strain specific qPCR. **Supplementary Figure 21.** Strain specific PCR. **Supplementary Figure 22.** Correlation analysis between the qPCR estimated copies of *L. rhamnosus* genome and *L. rhamnosus* relative abundance obtained via 16S compositional analysis. **Supplementary Figure 23. ***L. rhamnosus* and *S. thermophilus* show a similar. **Supplementary Figure 24.** Correlation between of *L. rhamnosus* and *S. thermophilus*. **Supplementary Table S1.** Protocol deviations. **Supplementary Table S2.** Demographic characteristics. **Supplementary Table S3.** Overview of the successful microbiota composition determination. **Supplementary Table S4.** Successful samples and success rate per trial phase. **Supplementary Table S5.** Differential abundance analysis, species. **Supplementary Table S6.** Differential abundance analysis, pathways. **Supplementary References.**

**Additional file 2: Supplementary Figure S16.** Overview of the microbiota composition through time per subject at phylum level.

**Additional file 3: Supplementary Figure S17.** Overview of the microbiota composition through time per subject at family level.

**Additional file 4.** CONSORT flow diagram.

**Additional file 5: Supplementary Figure S19.** Taxonomic composition at genera level of the *Peptostreptococcaceae* family thoughout the whole trial for subject VOL01, VOL03, VOL06, VOL08, VOL14, VOL14.

**Additional file 6. ***L. rhamnosus* enumeration via qPCR and calculation of the ileostomy effluent microbial density using the 16S metataxonomic data.

## Data Availability

The datasets supporting the conclusions of this article are available in the DANS repository, on the following doi: 16S 10.17026/dans-xhw-dhpr, Metatranscriptome 10.17026/dans-xvh-yww8, Permeability test 10.17026/dans-xnp-nkq8, Urine Metabolites: 10.17026/dans-2ad-cx47, Short Chain Fatty Acid profiling 10.17026/dans-z3q-vvz4. The supplementary 16S dataset used for the microbial density calculation is available: https://www.ncbi.nlm.nih.gov/bioproject/857869
