# Supplementary material for: Endogenous small intestinal microbiome determinants of transient colonisation efficiency by bacteria from fermented dairy products: a randomised controlled trial
Source: Microbiome. 2023 Mar 7;11:43. doi: 10.1186/s40168-023-01491-4 (PMC9990280; doi:10.1186/s40168-023-01491-4)
Supplement: Supplementary file 2 — Additional file 1: Supplementary Methods. Recruitment criteria, Gastrointestinal permeability, Short Chain Fatty Acids, Intestinal Microbiota composition, Metatranscriptomics, Urine metabolites profiling, Ileostomy effluent bacterial density calculation, Data mining and statistics. Supplementary Results. Gastrointestinal permeability and SCFA. Supplementary Figure 1. Microbial composition profiles comparison. Supplementary Figure S2. Type and severity of gastrointestinal symptoms. Supplementary Figure S3. Short chain fatty acid concentration in ileostomy effluents. Supplementary Figure S4. Gastro-intestinal permeability. Supplementary Figure S5. Short-term gastro-intestinal permeability. Supplementary Figure S6. Overall and longitudinal microbiota composition analysis. Supplementary Figure S7. Beta diversity of ileostomy effluent microbiota, intra and inter subject; Supplementary Figure S8. Carry-over effect analysis. Supplementary Figure S9. Alpha diversity of ileostomy effluent microbiota. Supplementary Figure S10. Beta diversity of the ileostomy effluent microbiota. Supplementary Figure S11. Core-microbiome. Supplementary Figure S12. L. rhamnosus relative abundance and microbial density in ileostomy samples. Figure S13. Inter subject differences functional metatranscriptome mapping analysis. Supplementary Figure S14. Intervention products effects on the functional metatranscriptome mapping of the ileostomy effluent. Supplementary Figure S15. Analysis of the relashionship between Peptostreptococcaceae and urine microbial metabolites generated through bacterial proteolytic fermentation. Supplementary Figure S18. Taxonomic composition at genera level of the Peptostreptococcaceae family per volunteer. Supplementary Figure 20. Strain specific qPCR. Supplementary Figure 21. Strain specific PCR. Supplementary Figure 22. Correlation analysis between the qPCR estimated copies of L. rhamnosus genome and L. rhamnosus relative abundance obtained via 16S compositional analysi [file 40168_2023_1491_MOESM1_ESM.pdf]

Supplementary materials for:

Endogenous small intestinal microbiome determinants of transient colonization efficiency by bacteria from fermented dairy products; a randomized controlled trial

Edoardo Zaccaria*,[1,2]

Tim Klaassen*,[2,3],

Annick M.E. Alleleyn*,[3]

Jos Boekhorst[1]

Tamara Smokvina[4]

Michiel Kleerebezem[#,1, §]

Freddy J. Troost[#,2,3]

* These authors contributed equally

# These authors contributed equally

1. Host Microbe Interactomics Group, Wageningen University & Research, De Elst 1, 6708WD Wageningen, The Netherlands,
2. Food Innovation and Health, Center for Healthy Eating and Food Innovation, Maastricht University,5911AA Venlo, The Netherlands
3. Division of Gastroenterology-Hepatology, Department of Internal Medicine, School of Nutrition and Translational Research in Metabolism (NUTRIM), Maastricht University Medical Center+, P.O. Box 5800, 6202AZ Maastricht, The Netherlands
4. Danone Research, Av. De la Vauve, 91767 Palaiseau, France

Supplementary Methods

Recruitment criteria

Inclusion criteria:

- Surgical procedure to create ileostomy at least 3 years prior to participation,
- Age between 18 and 70 years,
- Body mass index (BMI) between 18 and 28 kg/m2.

Exclusion criteria:

- History of chronic or severe disease,
- Use of medication or supplements influencing study endpoints within 14 days prior to participation,
- Administration of investigational drugs which interfere with this study,
- Surgery requiring general anaesthesia four weeks prior to participation,
- Known lactose intolerance or suspected allergy or hypersensitivity to any component of the study products,
- Severe gastrointestinal symptoms,
- Removal of more than 15 cm of the ileum during or at any moment after the colectomy procedure,
- History of abdominal surgery interfering with gastrointestinal function (other than colectomy),
- Self-admitted HIV-positive status,
- Consumption of probiotic or prebiotic supplements or pre- and probiotics containing food products four weeks prior to participation,
- Use of antibiotics four weeks prior to participation,
- Known pregnancy or lactation,
- Abuse of alcohol (>20 units per week) or drugs,
- Blood donation within 3 months before participation,
- History of any side effects towards intake of pro- or prebiotic supplements of any kind,
- Prohibited use of pro-, pre- or symbiotic during the study period and three months prior to participation (a list of forbidden products was provided).

Gastrointestinal permeability

At the start and end of each intervention period, on the day prior to visiting the testing facility, gastrointestinal (GI) permeability was assessed by a multi-sugar test that was completed at home. The procedure was adapted from Van Wijck et al. [1] to accommodate the anatomical difference, i.e. absence of a colon, of the subjects. After an overnight fast, subjects emptied their bladder and ingested a 250 mL tap water solution of a multi-sugar mix (1 g sucrose [Van Gilse, Dinteloord, the Netherlands], 0.5 g L-Rhamnose [Danisco Sweeteners, Thomson, IL, USA], and 1g lactulose [Centrafarm, Etten-Leur, the Netherlands]). Subsequently, subjects collected their urine for 5 hours post-ingestion in two fractions; the first fraction containing the 0-2 h urine output and a second fraction containing the 2-5 h urine output. During the 5h collection period, subjects were not allowed to ingest any foods or drinks, except water. The subjects handed in their collected urine samples on the next day when they visited the testing facility. At the testing facility, total urinary output volume of the 0-2h and 2-5h sample were measured, and 2 mL aliquots of the urine samples were stored at -80 ˚C until the day of analysis. Quantification of 0-5h urinary excretion of the three different ingested sugars by HPLC-MS [1, 2], enables the measurement of gastroduodenal- and SI permeability respectively [2]. Gastroduodenal and small intestinal permeability were reflected by the 0-5 urinary sucrose excretion and the lactulose and L-Rhamnose (L/R) ratio, respectively [2]. The L/R ratio was also assessed in the 0-2h urine excretion samples, as some studies suggest that this fraction provides a superior assessment of SI permeability in comparison to the 0-5 h fraction in healthy individuals with an intact colon. One subject did not comply to the instruction for the intestinal permeability test and could therefore not be included in this analysis.

Short Chain Fatty Acids

Concentrations of acetic acid, propionic acid, and butyric acid were determined in the 15 mL effluent samples collected for this purpose during the test days. For analyses 0.2 – 0.5 g of effluent was added into a 10 mL tube (Greiner screw cap tube, Sarstedt, USA), already containing 5 mL ethanol:water mixture (70:30 v/v). After thorough mixing undissolved material was removed by centrifugation (10’, 4000xg, 4 ˚C) and 500 µL of the transferred to a clean tube and centrifuged to remove remaining insoluble materials (15’, 8000xg, 4 ˚C). Finally, 75 µL of the liquid was carefully removed and mixed with 75 µL of water and in this final solution SCFAs were quantified using gas chromatography-mass spectrometry as previously described [3]. SCFA concentrations were expressed per gram of ileostomy effluent (wet weight).

Urine metabolites profiling

Metabolomics profiling was conducted using ultra-high-performance liquid chromatography-tandem mass-spectrometry by the metabolomics provider Metabolon Inc. (Morrisville, USA) on first-morning urine samples. The metabolomic dataset measured by Metabolon includes 873 known metabolites containing the following broad categories – amino acids, peptides, carbohydrates, energy intermediates, lipids, nucleotides, cofactors and vitamins, and xenobiotics. These include metabolites of established microbial origin. Data extraction, compound identification, and data processing were performed by Metabolon Inc. Compounds were identified by comparison to library entries of purified and authenticated standards. Metabolites were quantified by measuring the area-under-the-curve of the chromatographic peak and their abundance data were normalized by median scaling and missing values were imputed with half of the sample set minimum.

Ileostomy effluent bacterial density calculation

Subjects consumed a standardized breakfast with a single dose of *L. rhamnosus* CNCM I-3690 product. Subsequently, complete ileostomy effluent output has been collected, over time intervals of 4 hours during the first 12 hours following consumption of the products. These samples were used to assess the population size by qPCR, as well as the corresponding effects on SI microbiota composition via 16S analysis. Combining the results, we estimated the bacterial density in the ileostomy effluents. The specificity of the primers used (FOR AS113: GTGACAACCGCAATCACTTG, REV AS114: TATCGGTGCCATTGAGTGAA), targeting a gene encoding a putative transcriptional regulator of the Cro/CI family, was verified both in silico via BLAST search paying particular attention to align the sequences with genomes of bacterial genera typically found in ileostomy effluents, and in vitro performing PCR on DNA extracted from ileostomy effluents sampled prior L. rhamnosus product consumption

Supplementary Results

Gastrointestinal permeability and SCFA

To assess the impact of the product interventions on gastrointestinal permeability in the different regions of the gastroduodenal and small intestinal tract, the urinary recovery of an orally administered sugar mixture was determined over the first 5 hours post-consumption, in two intervals 0-2h and 2-5h, in all subjects with the exception of one participant, who did not comply to the permeability assessment protocol (see Materials and methods). Recovery in 0-5h urine of lactulose, sucrose, and L-Rhamnose and the Lactulose/L-Rhamnose ratio before and after each of the intervention periods (Supplementary figure 2, Supplementary figure 3) were determine. Based on a mixed model analysis, no statistically significant differences were found for lactulose recovery ($p = 0.454$), sucrose ($p= 0.256$), L-Rhamnose ($p= 0.220$), Lactulose/L-Rhamnose ratio ($p= 0.986$) between the various interventions as well as in the short-term Lactulose/L-Rhamnose ratio recovered in 0-2h urine output ($p= 0.322$; Supplementary figure 4). The short chain fatty acids propionic acid, acetic acid and butyric acid in ileostomy effluents were determined at the start and end day of each of the intervention periods (Supplementary figure 3) to assess the effect of the interventions on SCFA profiles. Based on a mixed model analysis, no statistically significant differences were found for propionic acid ($p = 0.772$), acetic acid ($p= 0.184$), or butyric acid ($p= 0.651$) between the various interventions (Supplementary figure 3).

Supplementary Figures

Supplementary Figure 1, microbial composition profiles comparison

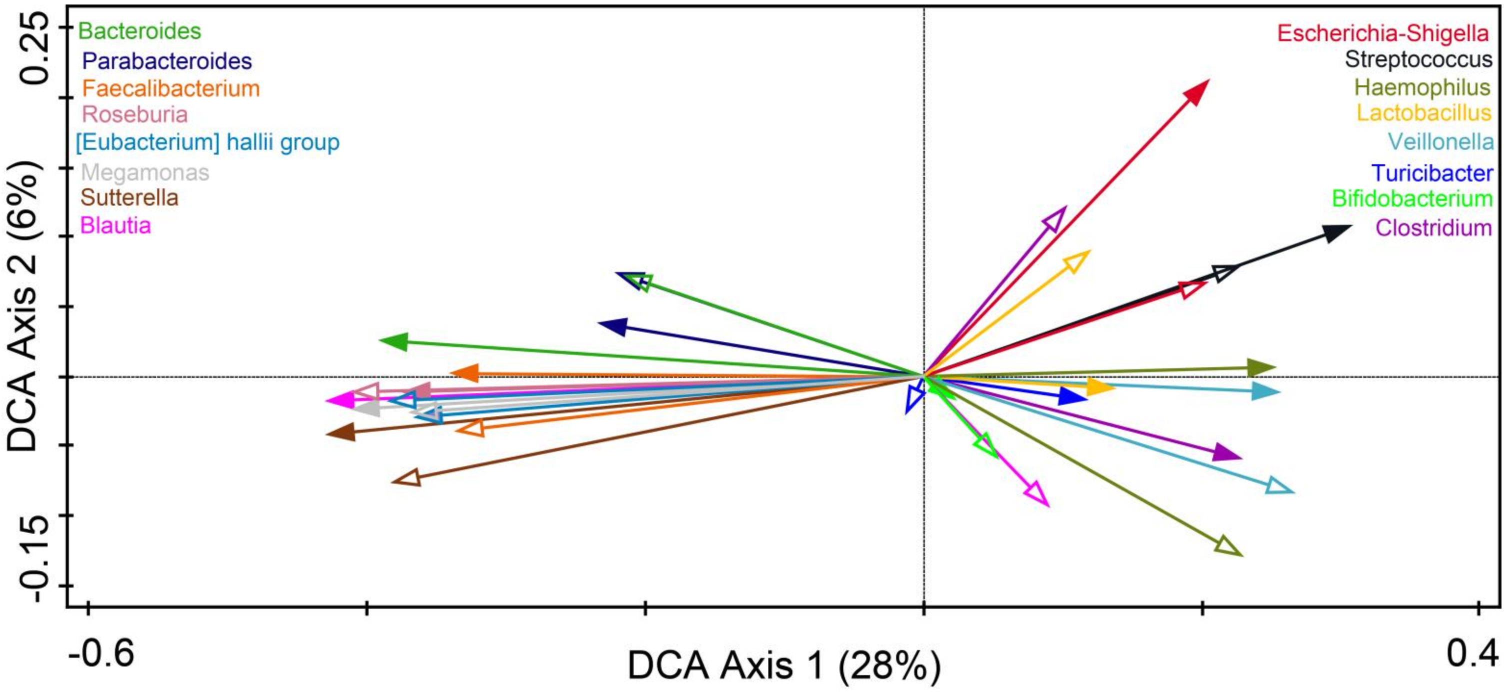

Supplementary Figure 1: DCA plot of 16S relative abundance on the genus level. The ordination space was determined by the genus relative abundance as determined through 16S amplicon sequence. The 16 most abundant taxa identified in both 16S amplicon sequencing and metatranscriptomics (of the 18 genera making up the first 90% of the relative abundance in metagenomics, 16 are found in the 34 genera making up the first 90% of the 16S dataset) were plotted as supplementary variables, with closed arrows representing 16S and open arrows metatranscriptomics, and arrow colors indicating genus (color legend in the figure). The plots shows substantial overlap between the compositional profiles, as indicated by arrows with same color pointing in similar directions.

Supplementary Figure 2, type and severity of gastrointestinal symptoms

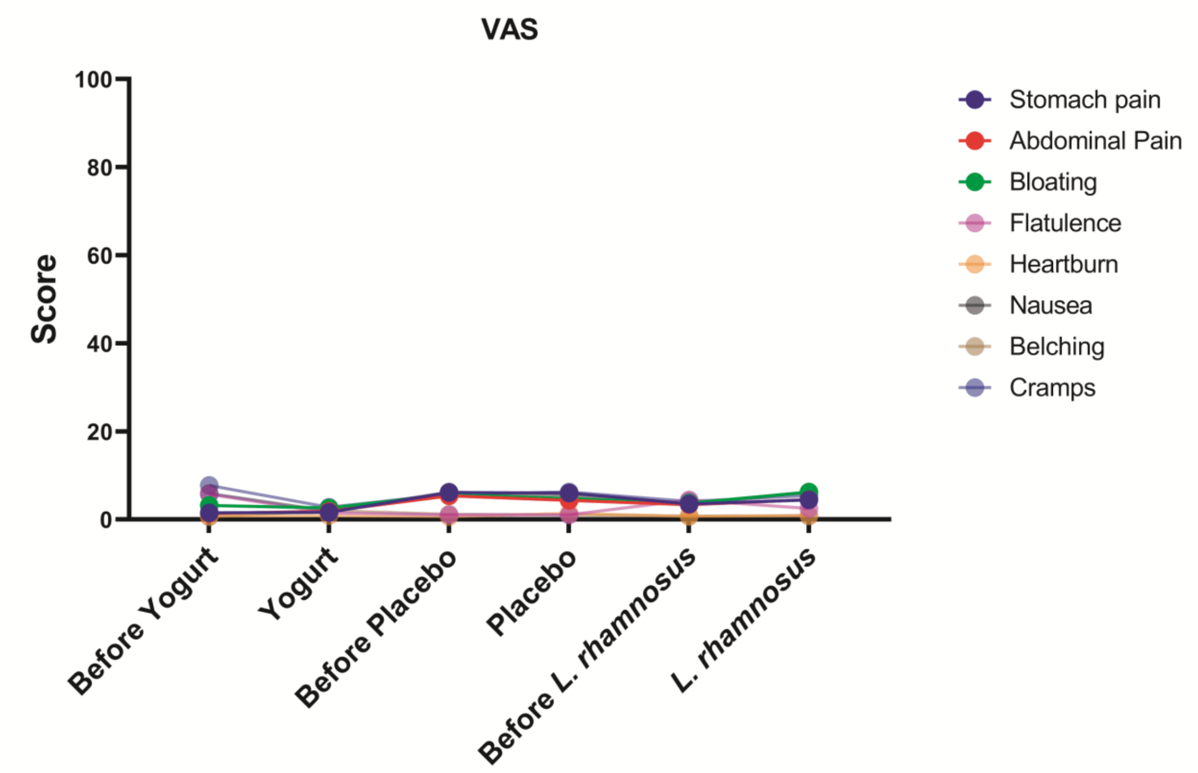

Supplementary Figure 2: Type and severity of gastrointestinal symptoms was not altered by consumption of the intervention products. Visual analogue scale (VAS) scores, measured on a 0-100 mm scale for stomach pain, abdominal pain, bloating, flatulence, heartburn, nausea, belching and cramps recorded before and after each intervention period was used to assess the type and severity of gastrointestinal symptoms in the subjects. Based on a Friedman test on differences After-Before intervention, no statistically significant differences were found; stomach pain, (p: 0.861); abdominal pain, (p: 0.566); bloating, (p: 0.901); flatulence, (p: 0.455); heartburn, (p: 0.9702); nausea, (p: 0.704); belching (p: 0.348); and cramps, (p: .0.936) between the interventions.

Supplementary Figure 3, short chain fatty acid concentration in ileostomy effluents

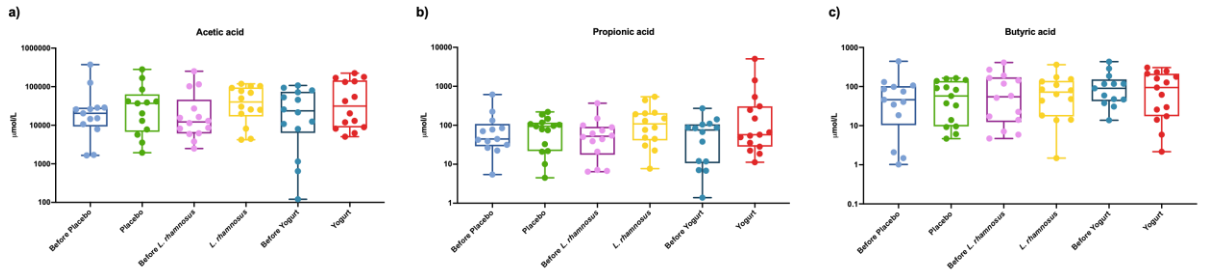

Supplementary Figure 3. Short chain fatty acid concentration in ileostomy effluents. Acetic acid (panel A), propionic acid (panel B) and Butyric acid (panel C) were measured in ileostomy effluent before and after each intervention. Based on a mixed model analysis, no statistically significant differences were found for propionic acid (p= 0.772), acetic acid (p = 0.184), or butyric acid (p= 0.651) between interventions.

Supplementary Figure 4, gastro-intestinal permeability

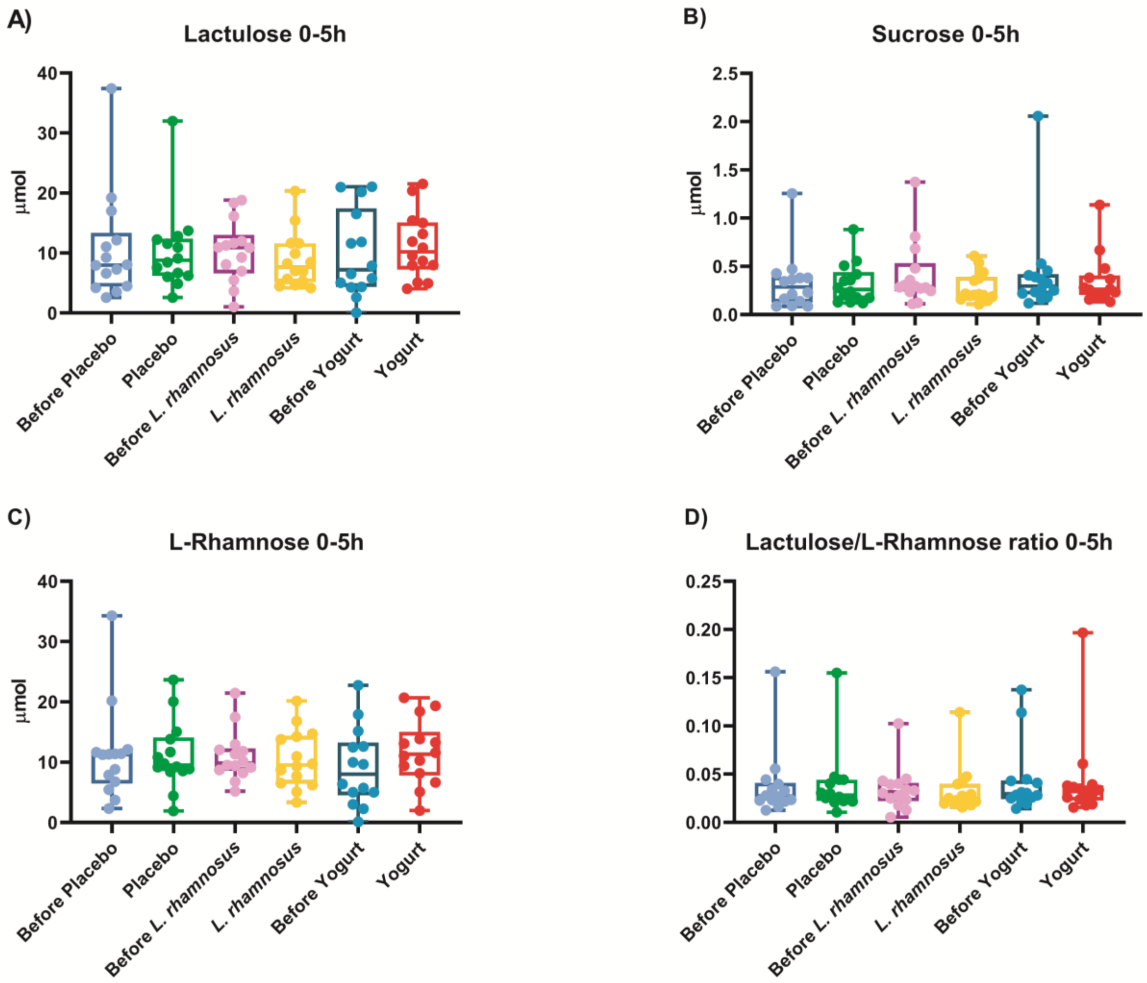

Supplementary Figure 4: Gastro-intestinal permeability was not affected by intervention products consumption. Gastro-intestinal permeability was measured by quantification of selected sugar recovered in 0-5h urine of lactulose (panel A), sucrose (panel B, L-Rhamnose (panel C) and Lactulose/L-Rhamnose ratio (panel D) before and after each intervention after ingestion of a sugar mix. Mixed model analysis revealed that there are no statistically significant differences between the interventions effect on permeability of lactulose (p = 0.454), sucrose (p= 0.256), L-Rhamnose (p= 0.220), L/R ratio (p= 0.986).

Supplementary Figure 5, short-term gastro-intestinal permeability

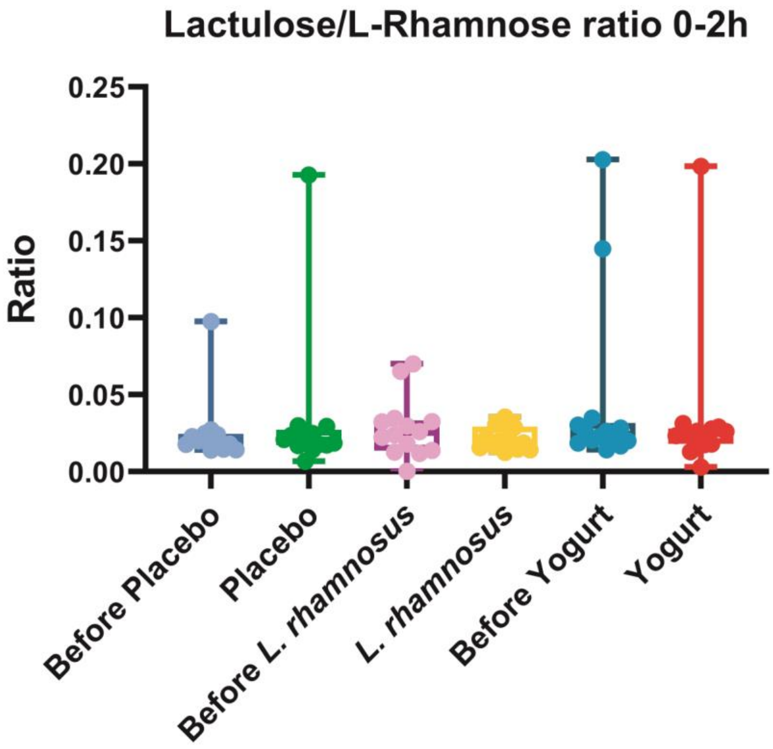

Supplementary Figure 5: Short-term gastro-intestinal permeability was not affected by intervention products consumption. Shor-term gastro-intestinal permeability was measured by quantification of Lactulose/L-Rhamnose ratio 0-2h urine before and after each intervention after ingestion of a sugar mix. Based on a mixed model analysis, no statistically significant differences were found for Lactulose/L-Rhamnose recovery ratio between the interventions (p= 0.322).

Supplementary Figure 6, Overall and longitudinal microbiota composition analysis

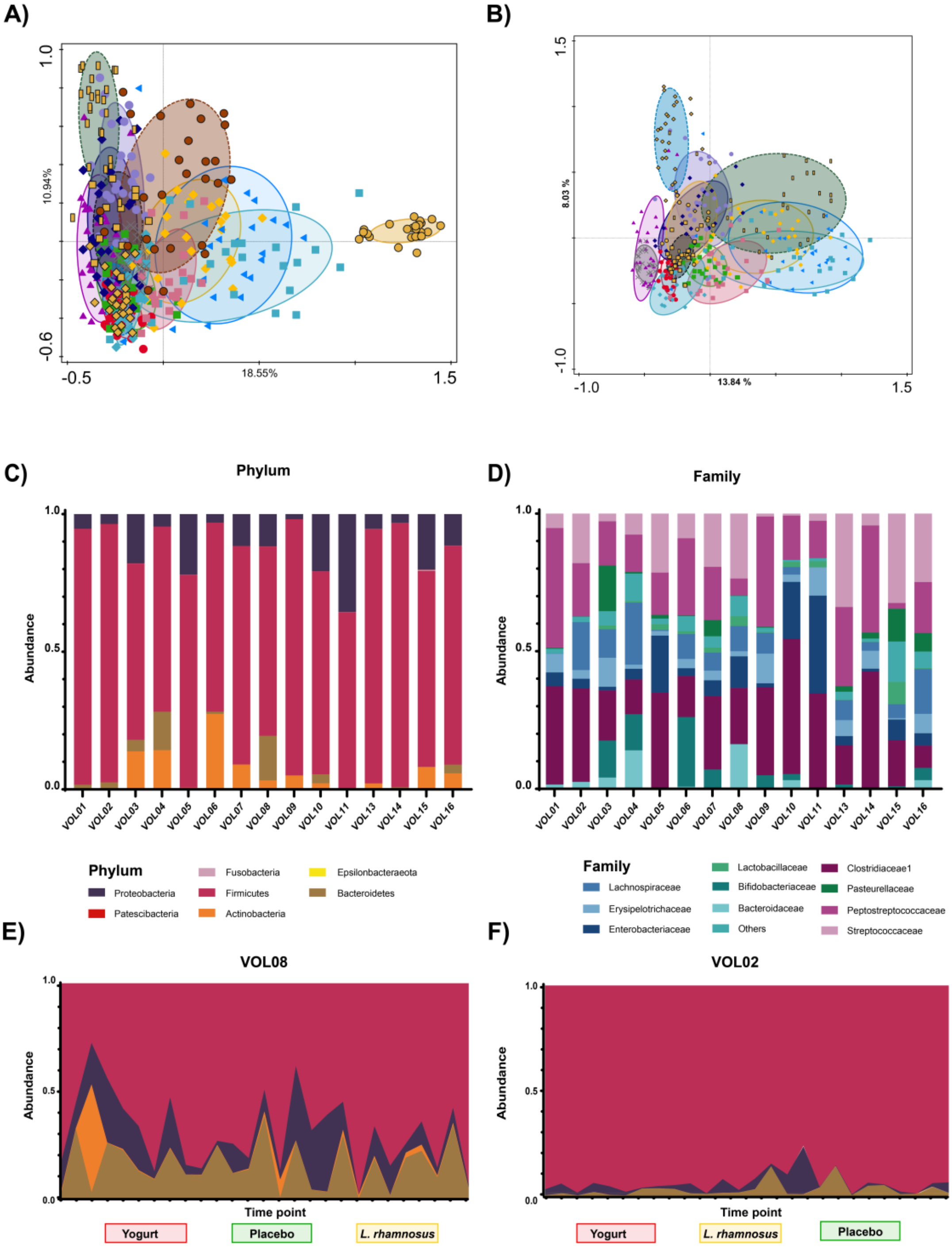

Supplementary Figure 6: Overall and longitudinal microbiota composition analysis. A general overview of the metataxonomic data distribution at OTU level presented via principal component analysis revealed that all samples obtained from one subject (in ochre) strongly deviated from the rest (panel A). This subject was the only individual with a Kock pouch rather than the standard ileostomy, which led us to exclude this subject in further analysis as a biological outlier. Redundancy analysis on the remaining samples revealed that 46.6% of the overall microbiota composition at species level was explained by subject ID (panel B, p=.001). The intra-subjects' variation is highlighted also in the general overview of the averaged microbiota composition per subject presented at phylum (panel C) and family (panel D) level. Two examples of the microbiota composition over the whole length of the trial illustrates the observed high (panel E) and low (panel F) microbiota composition fluctuation over time at phylum level.

Supplementary Figure 7, beta diversity of ileostomy effluent microbiota, intra and inter subject

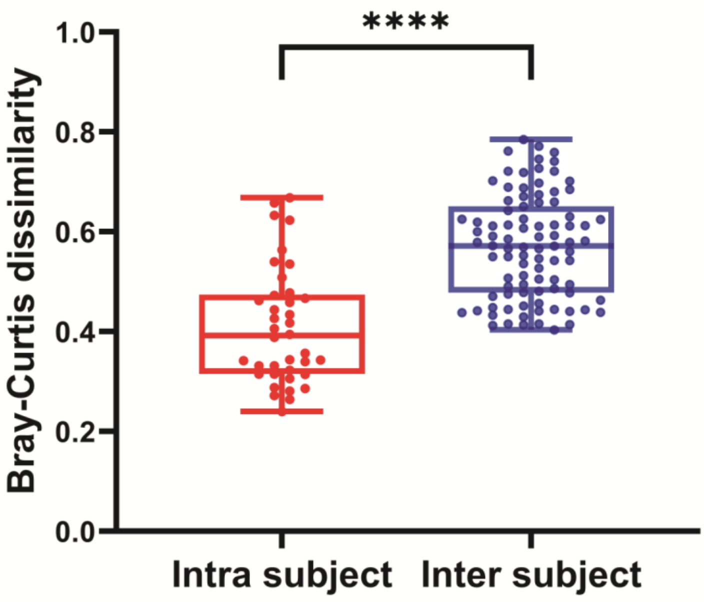

Supplementary Figure 7: beta diversity of ileostomy effluent microbiota, intra and inter subject comparison. The dissimilarity between samples intra and inter subjects was calculated via Bray-Curtis dissimilarity index throughout the study period and showed that a significantly higher degree of dissimilarities between subjects as compared to within subjects. Bray-Curtis distances intra and inter-subject, Mann-Whitney test, $p<.0001$.

Supplementary Figure 8, carry-over effect analysis

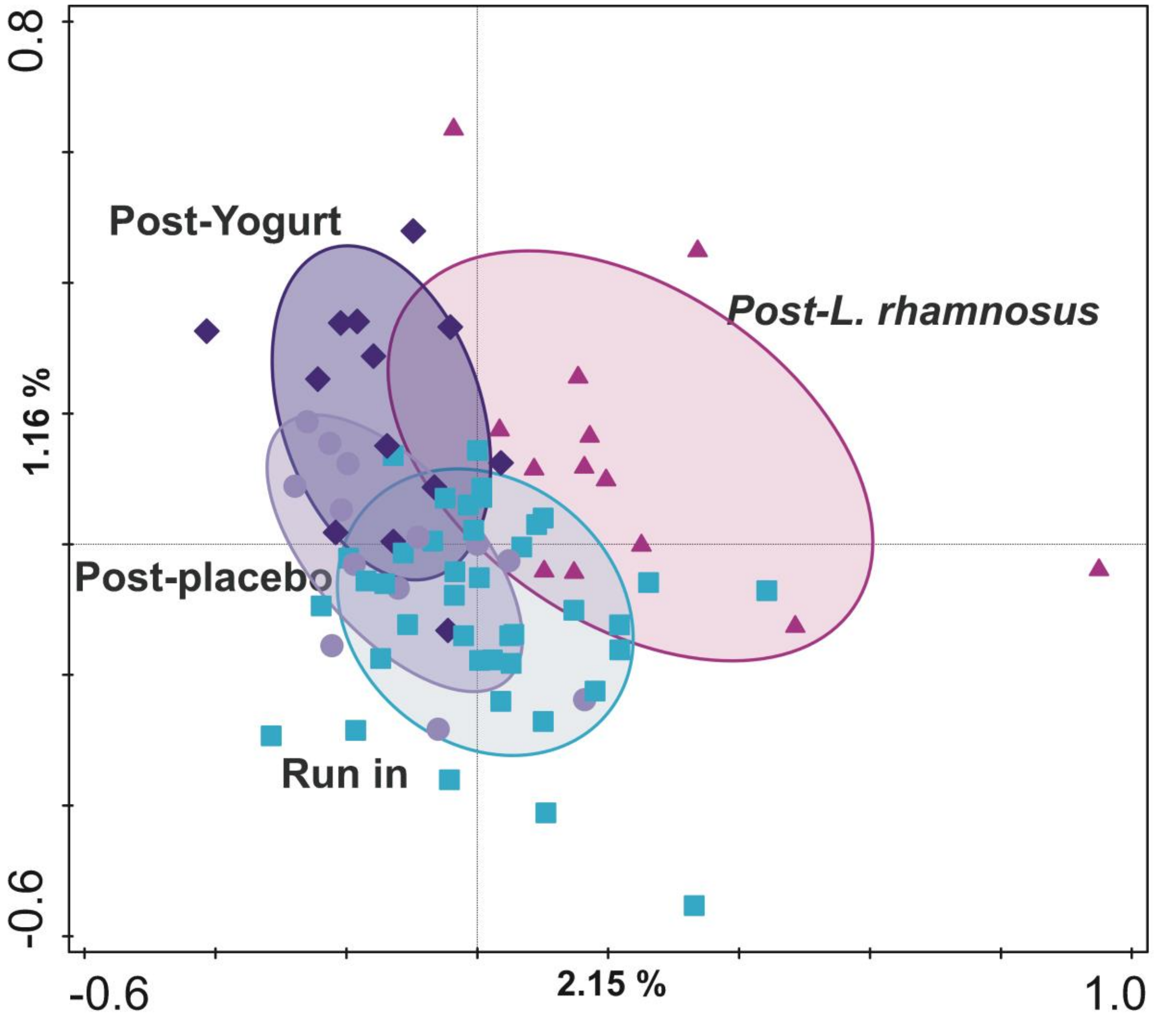

*Supplementary Figure 8: Carry-over effect analysis. The carry-over effect of the intervention was analysed by grouping the samples according to their preceding intervention (Post-*L. rhamnosus*, Post-Yogurt and Post-Placebo) and compared to the run-in samples of the subjects using a partial RDA (corrected for subject). The analysis failed to reveal differences between the sample-groups (explained variation: .00%, p=0.7253) and confirmed that the washout periods were long enough for the effluent microbiota composition to return to a state that is undistinguishable from the subject-specific baseline.*

Supplementary Figure 9, alpha diversity of ileostomy effluent microbiota

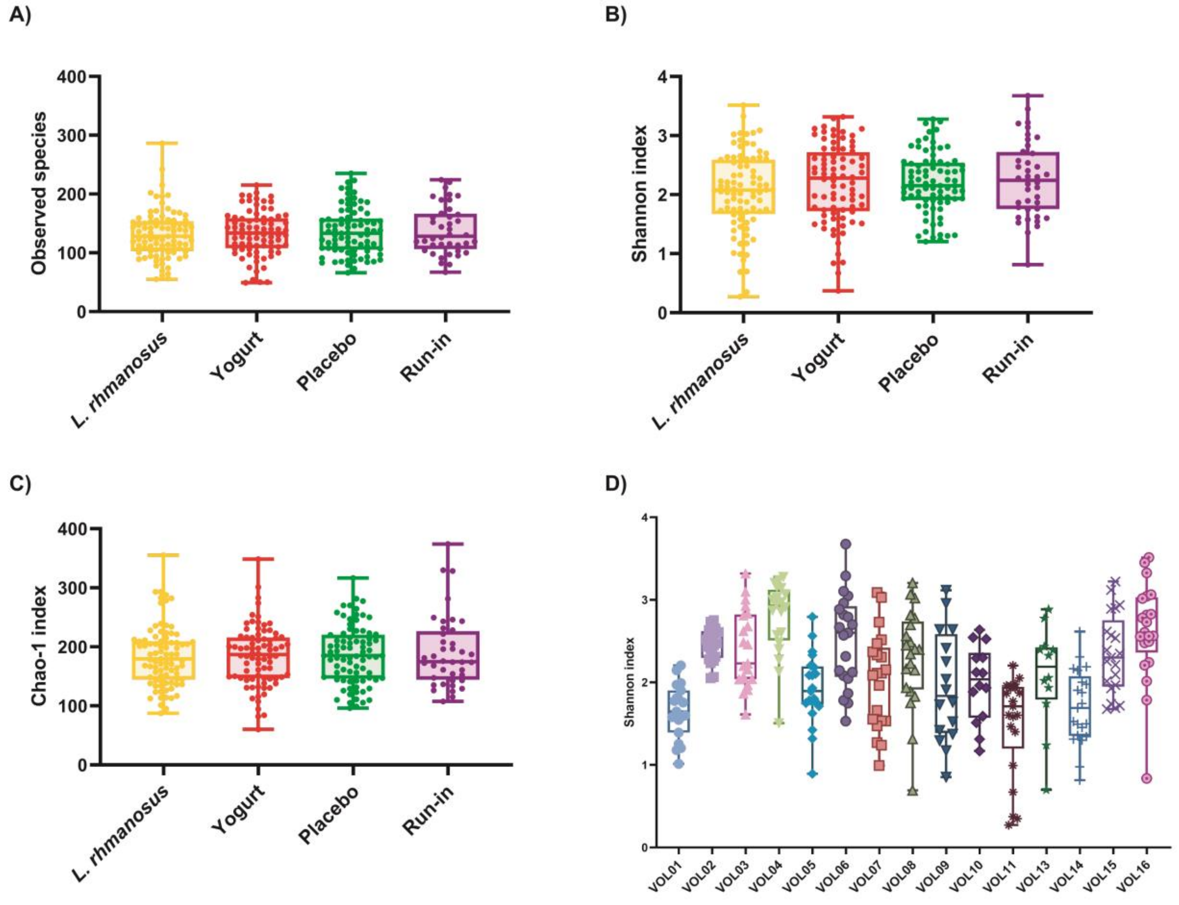

Supplementary Figure 9: alpha diversity of ileostomy effluent microbiota remained unchanged during the intervention periods compared with the Run-in. The effect of the intervention products on the alpha diversity of the ileostomy effluent microbiota was assess at species level using observed species (panel A), Shannon's (panel B) and Chao-1 (panel C) diversity index during intervention period and run-in periods. Ingestion of the products did not significantly alter the alpha diversity (ANOVA p value=0.7543, 0.2419 and 0.9066 respectively for pane A, B and C). Interestingly, following up analysis of the alpha diversity at species level revealed a high variability between subjects (panel D), Shannon index (ANOVA, p<.0001).

Supplementary Figure 10, beta diversity of the ileostomy effluent microbiota

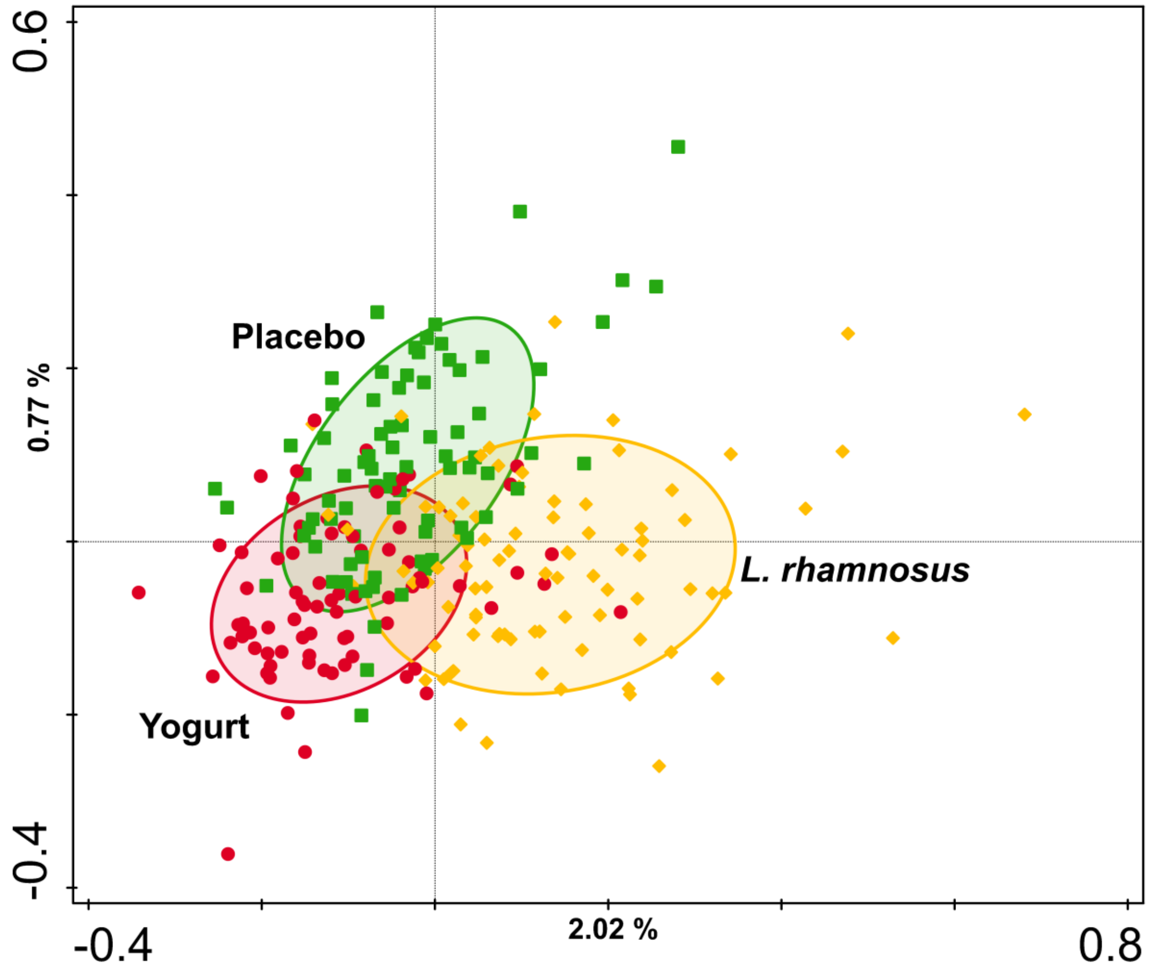

Supplementary Figure 10: the beta diversity of the ileostomy effluent microbiota during the intervention period is significant different. The distance-based partial redundancy analysis (subject ID used as covariant) revealed a significant difference between the beta diversity of the microbiota of ileostomy effluent during the consumption of the intervention products. Bray-Curtis dissimilarity, explained variation by the intervention 1.92%, p=.001. The three interventions indicated Yogurt (red), Placebo (green) and L. rhamnosus (yellow).

Supplementary Figure 11, core-microbiome

A)

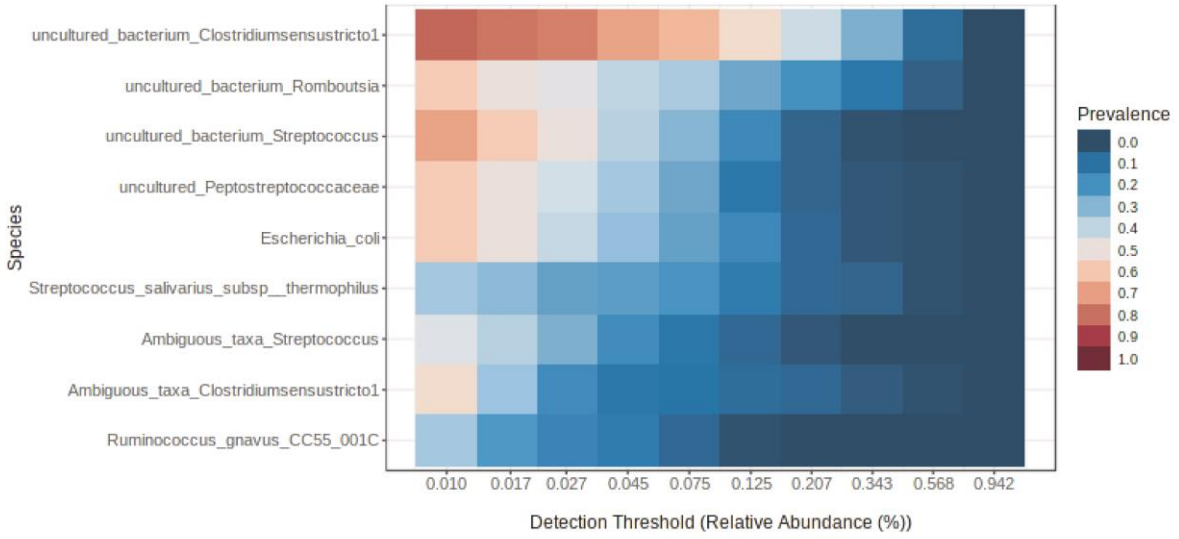

B)

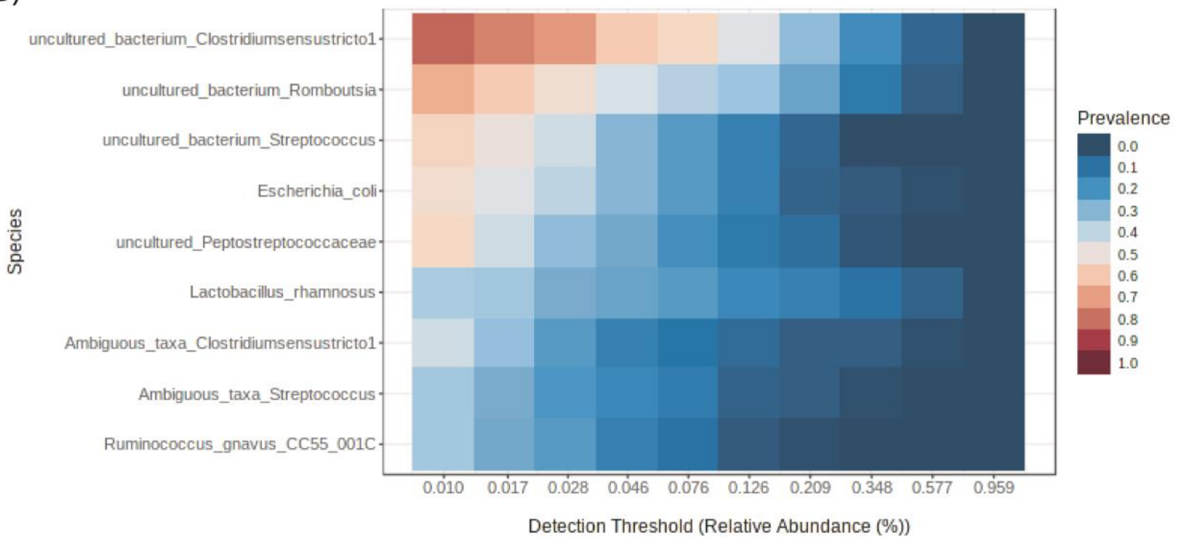

C)

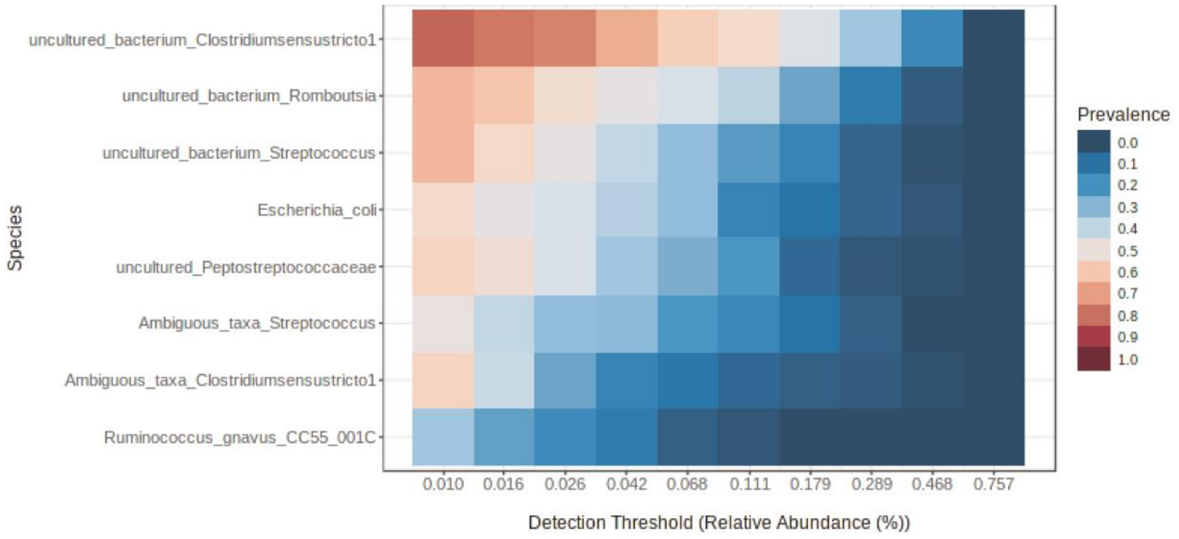

Supplementary Figure 11: core-microbiome analysis of the ileostomy effluent microbiota during intervention period. Core-microbiome analysis revealed that PDB appeared as members of the core-microbiome of the effluent samples during the respective intervention periods namely Yogurt (panel A), L. rhamnosus (panel B) and Placebo (panel C). To notice both L. rhamnosus and S. thermophilus become 6th member of the core-microbiome. The parameters utilized: sample prevalence, 30%; relative abundance, .01%.

Supplementary Figure 12, *L. rhamnosus* relative abundance and microbial density in ileostomy samples

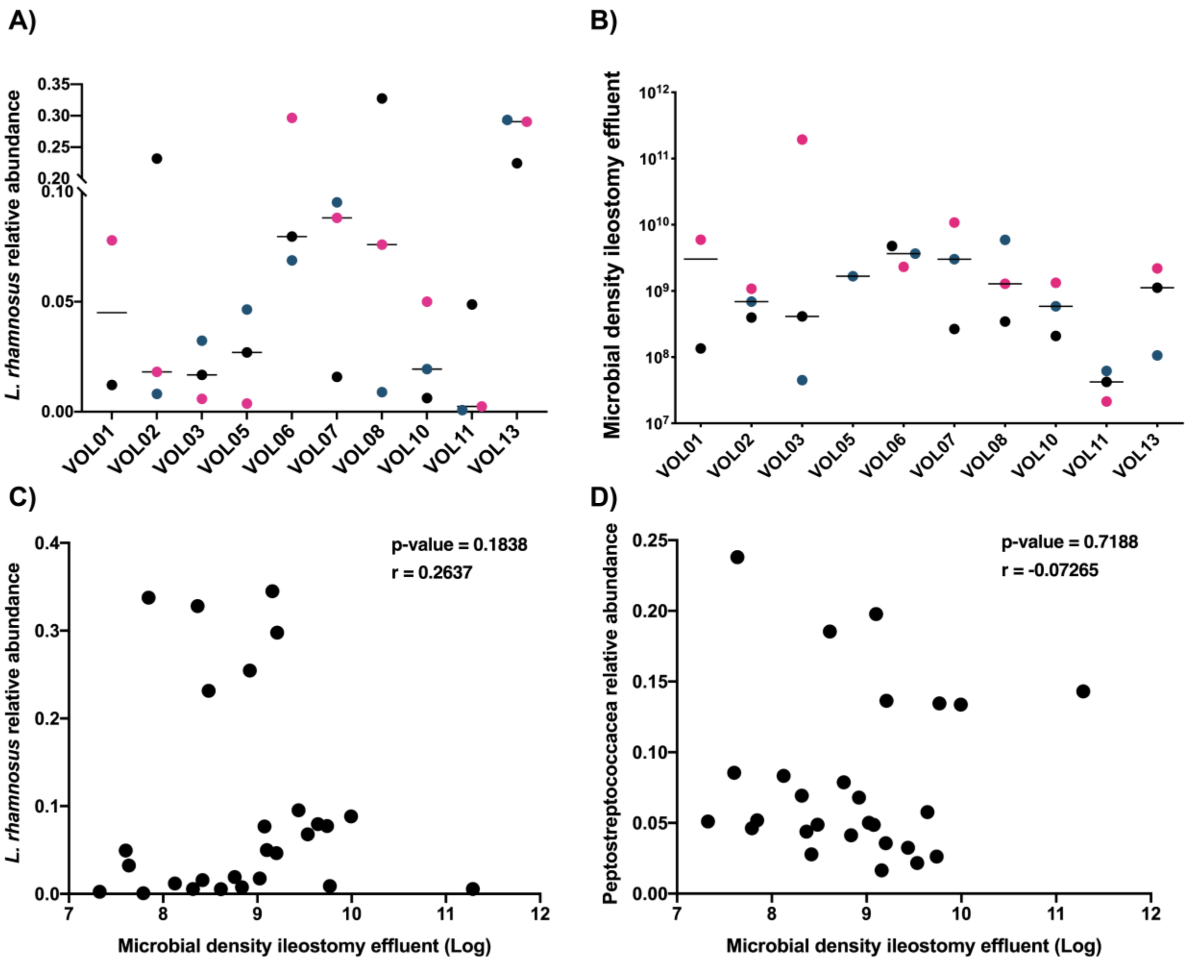

Supplementary Figure 12: microbial density does not correlate with pepetostreptococcacea nor with L. rhamnosus relative abundances. Subjects consumed a standardized breakfast with a single dose of L. rhamnosus CNCM I-3690 product, identical to those used in the 2-weeks intervention period. Subsequently, complete ileostomy effluent output has been collected, over time intervals of 4 hours during the first 12 hours following consumption of the products. These samples were used to assess the population size and qPCR, as well as the corresponding effects on SI microbiota composition via 16S analysis. Combining the results, we determined the bacterial density in the ileostomy effluents and we did not find any correlation between the ileostomy microbial density and the relative abundance of L. rhamnosus and/or of the peptostreptococcacea. The specificity of the primers used (FOR AS113: GTGACAACCGCAATCACTTG, REV AS114: TATCGGTGCCATTGAGTGAA), targeting a gene encoding a putative transcriptional regulator of the Cro/CI family, was verified both in silico via BLAST search paying particular attention to align the sequences with genomes of bacterial genera typically found in ileostomy effluents, and in vitro performing PCR on DNA extracted from ileostomy effluents sampled prior L. rhamnosus product consumption

Supplementary Figure 13, inter subject differences functional metatranscriptome mapping analysis

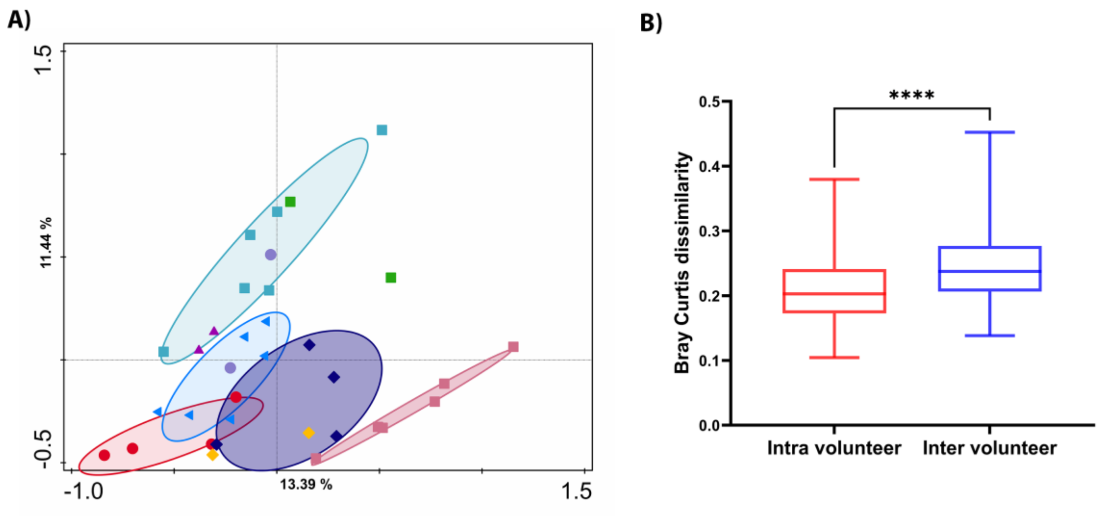

Supplementary Figure 13: Inter subject differences is the predominant source of variation of the functional metatranscriptome mapping dataset. Redundancy analysis of the overall microbial activity data distribution of the ileostomy effluents throughout the whole trial, clustered by subjects, revealing that 24.79% of the total data variation is due to the subject ID (panel A, p=.002). This result was supported by the higher Bray-Curtis dissimilarity between subjects compared with within subject (panel B), Mann-Whitney test, p<.0001

Supplementary Figure 14, intervention products effects on the functional metatranscriptome mapping of the ileostomy effluent

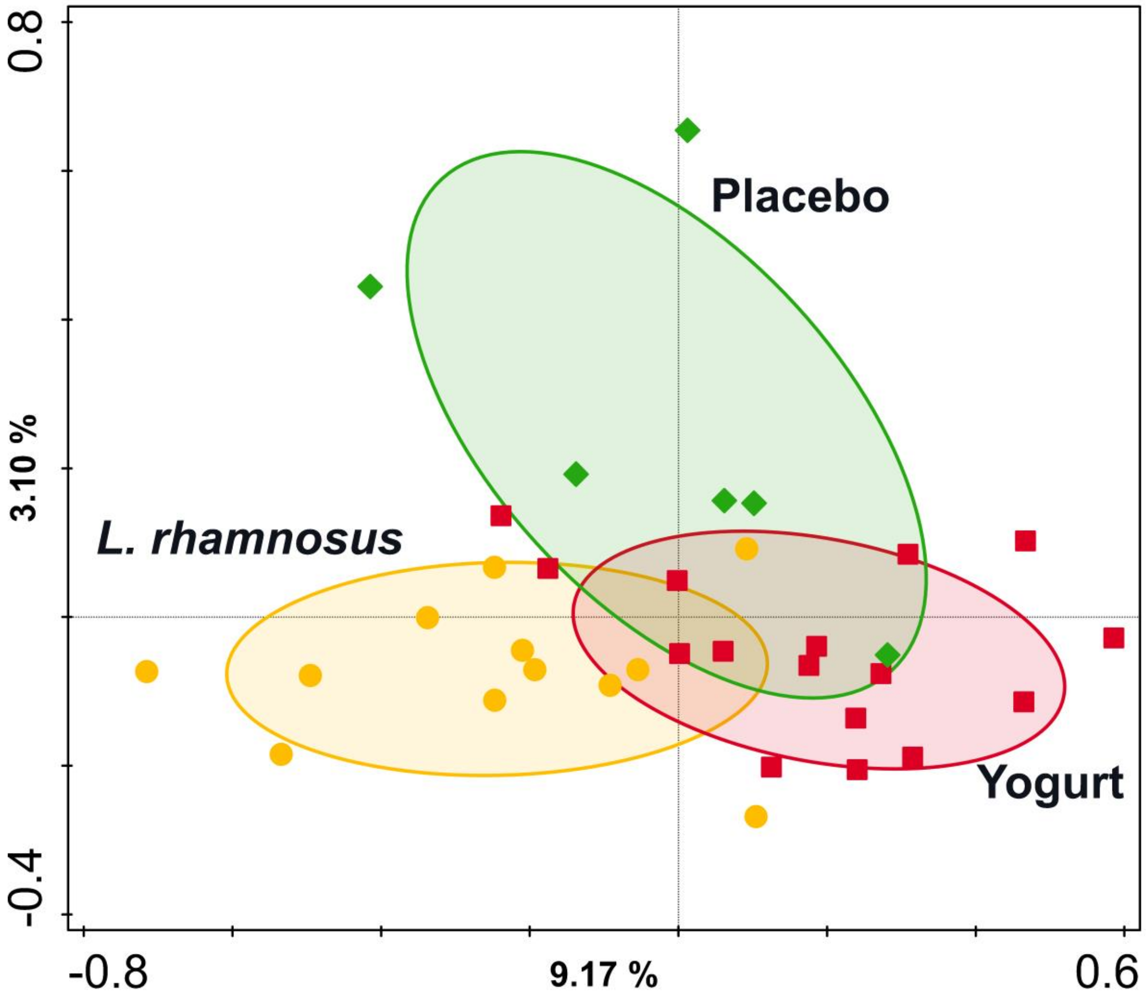

Supplementary Figure 14: intervention products effects on the functional metatranscriptome mapping of the ileostomy effluent. Redundancy analysis of the overall microbial functionality at pathway level was significantly affected by the products consumption. The interventions could explain 4.64 % of the overall variance in the FMM data (p=.042).

Supplementary Figure 15, analysis of the relashionship between *Peptostreptococcaceae* and urine microbial metabolites generated through bacterial proteolytic fermentation

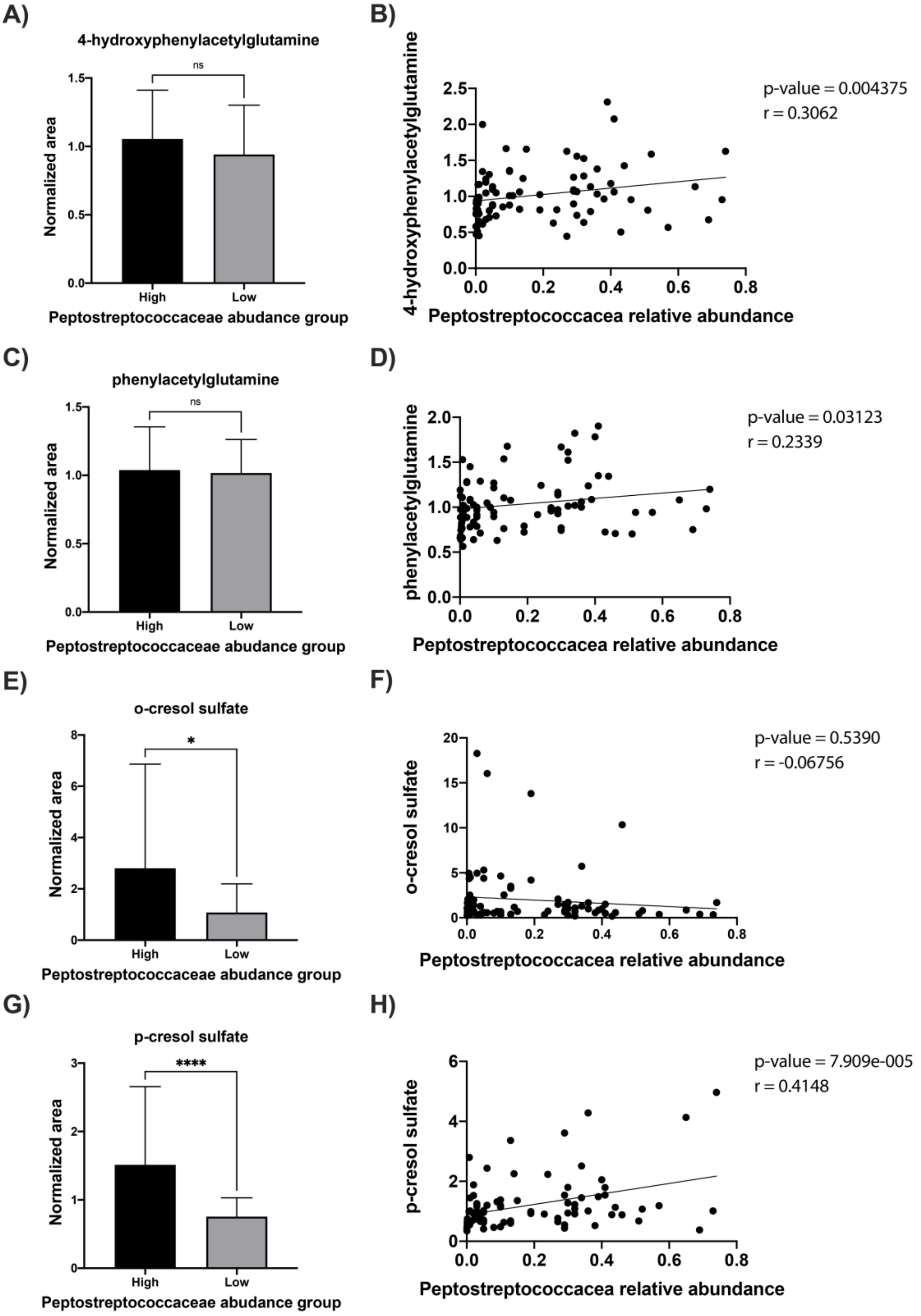

Supplementary figure 15: metabolites generated through proteolytic fermentation further support the link between Peptostreptococcaceae and acid-derived energy metabolism. Microbial metabolites generated through proteolytic fermentation, found in urine, positively correlates with Peptostreptococcaceae relative abundance (panel B, D, H Spearman rank) and it is found in significant higher amount in subjects with a high Peptostreptococcaceae

*abundance (panel E, G, high: Peptostreptococcaceae relative abundance > 0.15, Mann-Whitney test, * = p<.05, **** = p<.0001). Cresol is a methyl phenol produced via microbial degradation of tyrosine, while phenylacetylglutamine as well as 4-hydroxyphenylacetylglutamine result from glutamine conjugation of phenylacetic acid, which is almost exclusively derived from the microbial conversion of phenylalanine.*

Supplementary figure 16: overview of the microbiota composition through time per subject at phylum level.

Supplementary Figure 16: overview of the microbiota composition over the whole length of the trial at phylum level for each volunteer enrolled illustrates the observed high personalized microbiota composition and fluctuation whitin and among subjects.

Supplementary figure 17: overview of the microbiota composition through time per subject at family level.

Supplementary Figure 17: overview of the microbiota composition over the whole length of the trial at family level for each volunteer enrolled illustrates the observed high personalized microbiota composition and fluctuation whitin and among subjects. Taxa with less than 10 counts were merged.

Supplementary Figure 18, taxonomic composition at genera level of the Peptostreptococcaceae family per volunteer

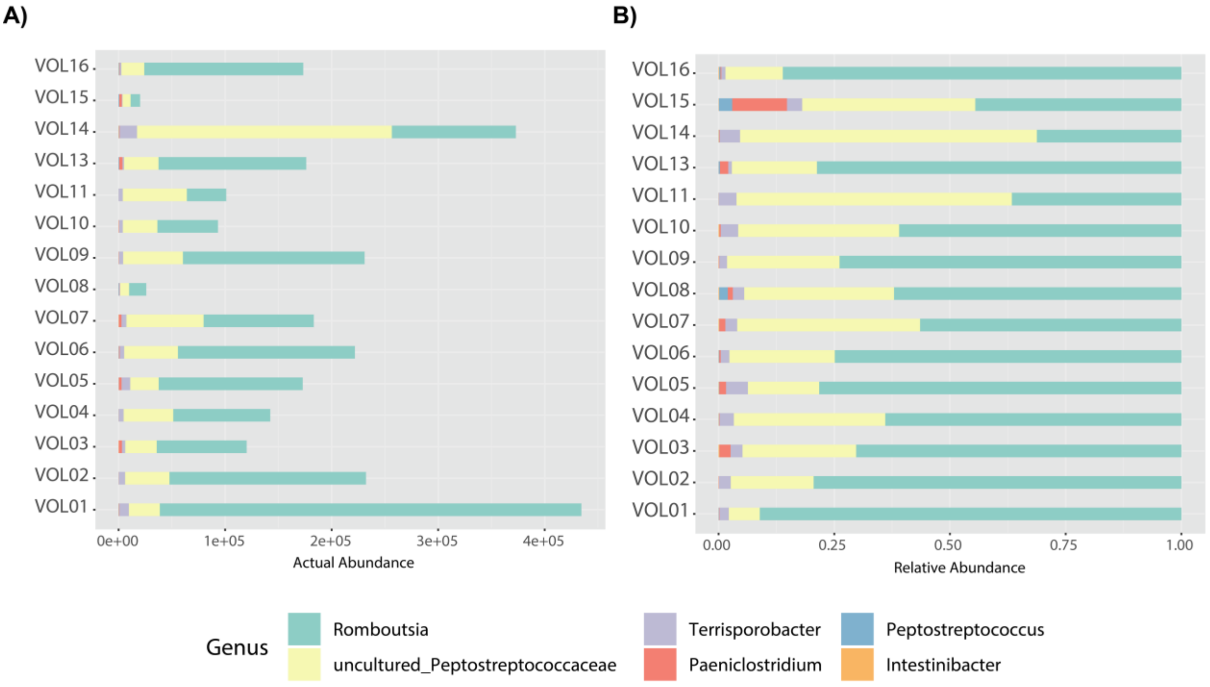

Supplementary Figure 18: overview of the Peptostreptococcacea composition averaged per volunteer enrolled illustrates the observed high personalized microbiota composition and fluctuation among subjects. Taxas with less than 5 counts were merged.

Supplementary Figure 19, taxonomic composition at genera level of the Peptostreptococcaceae family thoughout the whole trial for subject VOL01, VOL03, VOL06, VOL08, VOL14, VOL14

Supplementary Figure 19: overview of the Peptostreptococcacea composition over the whole length of the trial at genus level for selected volunteer enrolled illustrates the observed high personalized peptostreptococcacea composition and fluctuation whitin and among subjects. Taxa with less than 5 counts were merged.

Supplementary Figure 20, strain specific qPCR

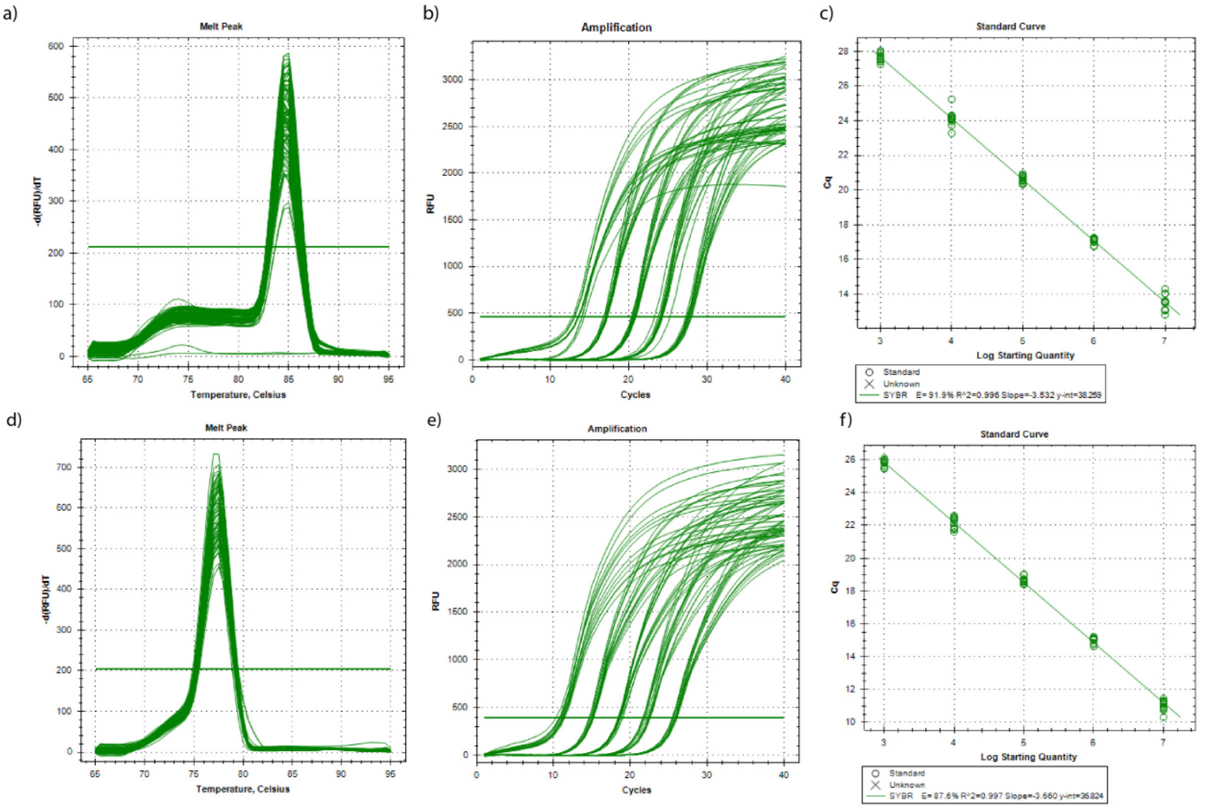

Supplementary Figure 20: Calibration curve on genomic DNA of L. rhamnosus CNCM I-3690 (a-c) and S. thermophilus CNCM I-1630 (d-f). For L. rhamnosus CNCM I-3690 strain-specif primers were used (FOR AS113: GTGACAACCGCAATCACTTG, REV AS114: TATCGGTGCCATTGAGTGAA) and the results indicate the producion of a single, specific products and a good linearity, precision and accuracy in quantified known amount of rhamnosus CNCM I-3690 genome. Similarly, for S. thermophilus CNCM I-1630 strain-specif primers (FOR OFF2540: CTATCGAACATTTACGAGCTG, REV OFF2541: GTATCTGTTGAAAGAGGTGTG) targeting the CRISPR reagion of the genome. Briefly serial diluted, known amount of rhamnosus CNCM I-3690 genome copies (10E+07-10E+03) were spiked in 3 ileostomy effluents collected prior products consumpion. The DNA was extracted and the qPCR was performed quadruplicate.The melting curves of the primers indicate that the reactions have produced single, specific products adn the limit of quantification was et to LOG3.

Supplementary Figure 21, strain specific PCR

a) b)

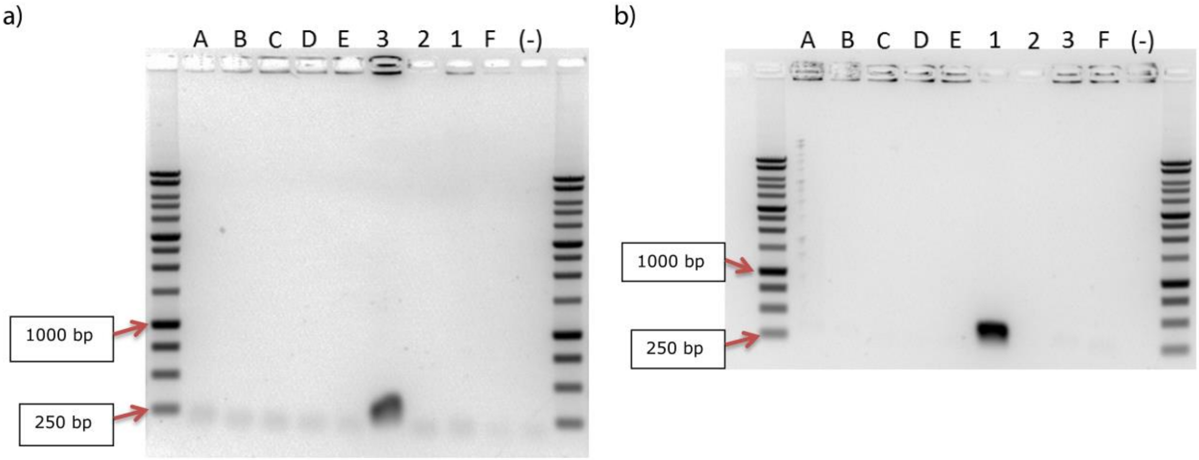

Supplementary Figure 21: electrophoresis DNA gel showing strain-specificity of the primers. To test the specificity in vitro, L. rhamnosus CNCM I-3690 strain-specif primers (FOR AS113: GTGACAACCGCAATCACTTG, REV AS114: TATCGGTGCCATTGAGTGAA) and S. thermophilus CNCM I-1630 strain-specif primers (FOR OFF2540: CTATCGAACATTTACGAGCTG, REV OFF2541: GTATCTGTTGAAAGAGGTGTG) were used in PCRs with ileostomy effluent DNA as template spiked with DNA derived from Streptococcus thermophilus CNCM I-1630(1), Lactobacillus bulgaricus CNCM I-1519 (2) and Lactobacillus rhamnosus CNCM I-3690 (3) and from the following 6 bacterial species isolated from human ileostomy effluent samples, Streptococcus mitis HSIS-M1 (A), Streptococcus bovis HSIS-B1 (B), Streptococcus salivarius HSIS-S1 (C), Streptococcus salivarius HSIS-S2 (D), Streptococcus salivarius HSIS-S3 (E), Streptococcus salivarius MS ileo (F).

Supplementary Figure 22, correlation analysis between the qPCR estimated copies of *L. rhamnosus* genome and *L. rhamnosus* relative abundance obtained via 16S compositional analysis

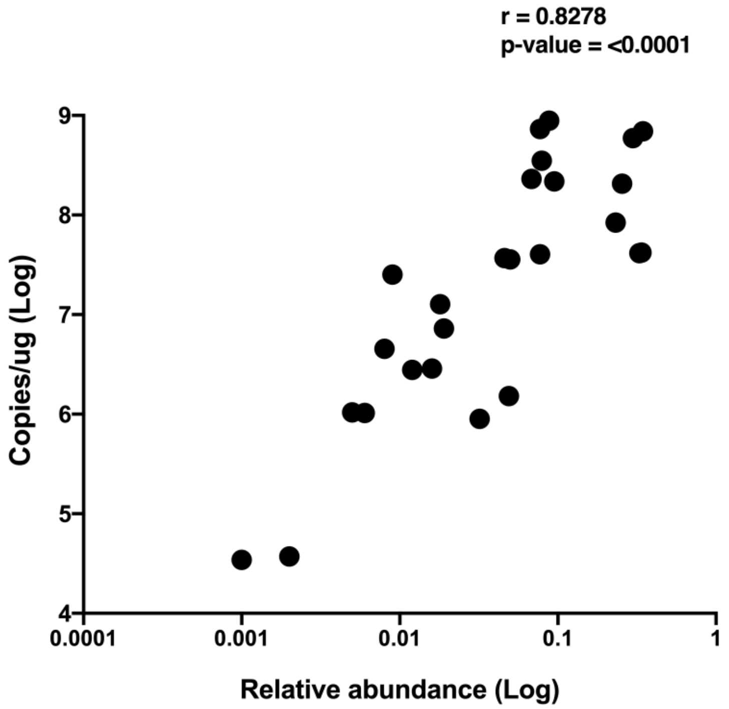

Supplementary Figure 22: Correlation (Spearman) analysis between the qPCR estimated copies of L. rhamnosus genome and L. rhamnosus relative abundance obtained via 16S compositional analysis. The correlation confirmed that the OTUs assigned by CLC workbench to L. rhamnosus well represents L. rhamnosus CNCM I-3690 in ileostomy samples. A sample was removed from the plot as considered outlier (relative abundance: 0.006, LOG copies/ng: $1.7x10^{9}$).

Supplementary Figure 23, *L. rhamnosus* and *S. thermophilus* show a similar

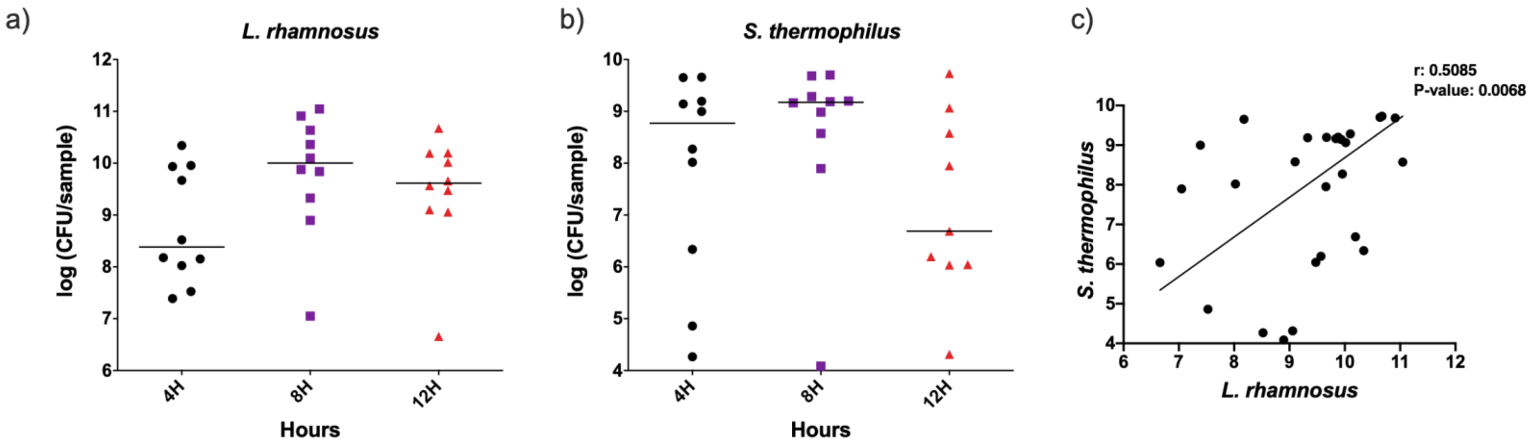

Supplementary Figure 23: qPCR estimated copies of L. rhamnosus CNCM I-3690 (a), S. thermophilus CNCM I-1630 (b) genome and their correlation (c, Spearman).Subjects consumed a standardized breakfast with a single dose of L. rhamnosus CNCM I-3690 product or, on a different day, of Yogurt (fermented by S. thermophilus CNCM I-1630 and L. bulgaricus CNCM I-1519) identical to those used in the 2-weeks intervention period. Subsequently, complete ileostomy effluent output has been collected, over time intervals of 4 hours during the first 12 hours following consumption of the products. These samples were used to assess the population size and qPCR. The specificity of the primers used for L. rhamnosus (FOR AS113: GTGACAACCGCAATCACTTG, REV AS114: TATCGGTGCCATTGAGTGAA) targeting a gene encoding a putative transcriptional regulator of the Cro/CI family and for S. thermophilus (FOR OFF2540: CTATCGAACATTTACGAGCTG, REV OFF2541: GTATCTGTTGAAAGAGGTGTG) targeting the CRISPR region of the genome were verified both in silico via BLAST search paying particular attention to align the sequences with genomes of bacterial genera typically found in ileostomy effluents, and in vitro performing PCR on DNA extracted from ileostomy effluents sampled prior to product consumption.

Supplementary Figure 24, correlation between of *L. rhamnosus* and *S. thermophilus*

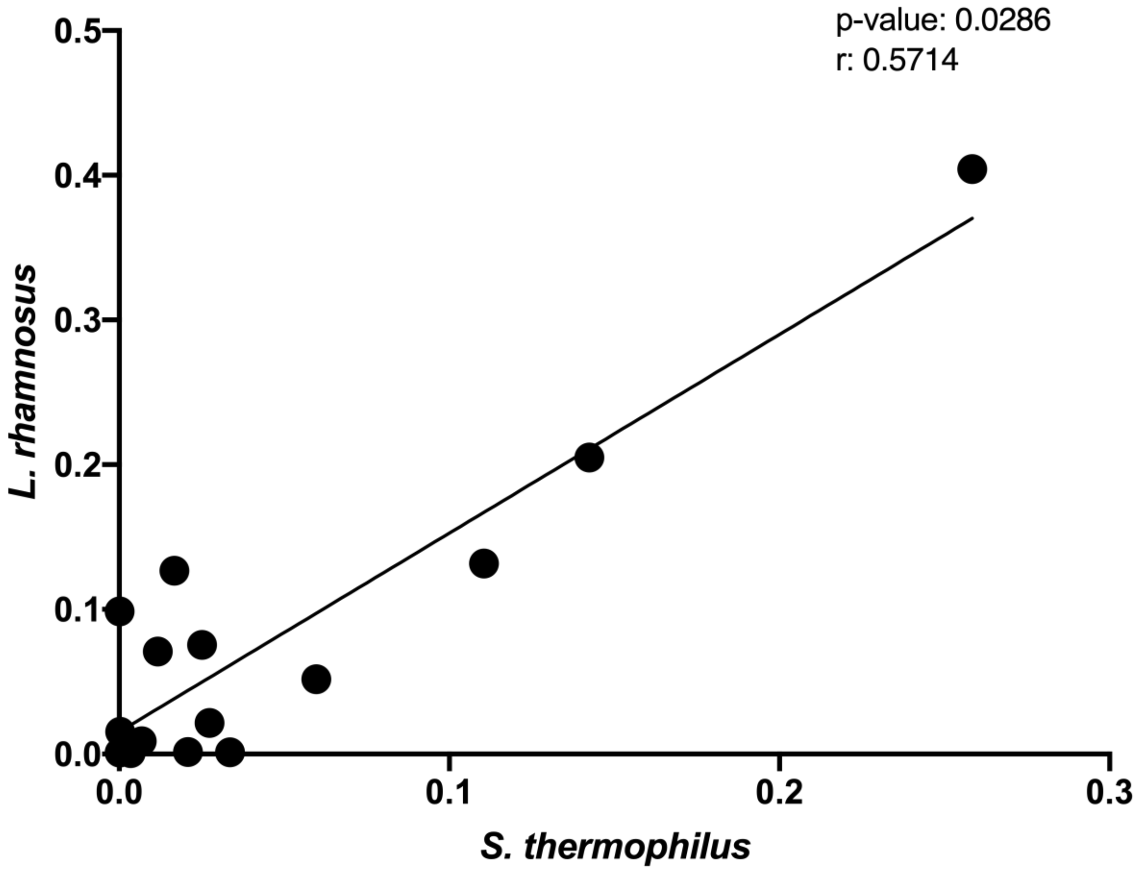

Supplementary Figure 24: correlation between the averages per volunteer of the relative abundance of L. rhamnosus and S. thermophilus.The analysis shows the congruent relative abundance of the two dominant PDB in individuals, which in combination with the highly comparable transit curves for these strains (see supplemental figure 23) indicates that the OTUs assigned to S. thermophilus represent an appropriate approximation for S. thermophilus CNCM I-1630.

Supplementary Tables

Supplementary Table 1, protocol deviations

Supplementary table 1: deviations from the protocol throughout the whole trial

Subject	Date of deviation	Kind of deviation	Comments
VOL08	Day 7, intervention 1	Deviation from product consumption (skipped one yogurt)	Abdominal pain and bloating
VOL12	Day 13, intervention 3	Deviation from product consumption (skipped one yogurt)	Forgot to drink the product
VOL15	Day 14, intervention 2	Deviation from product consumption (skipped one yogurt)	Accidentally threw out one of the yogurts
VOL15	Wash-out period 2	Deviation to diet restriction (ate one spoon of commercial yogurt	Stopped eating the yogurt after the 1st spoon

Supplementary Table 2, demographic characteristics

Supplementary table 2: demographic characteristics of the enrolled subjects (n=16)

**Characteristics**	**Mean [SD]**	**Min-max**	**Median**
Gender	10 Female [62,5%]	N/A	N/A
Age (yr)	49,2 [12,8]	24-62	53
Height (m)	1,72 [0,11]	1,56-1,94	1,70
Weight (kg)	73,40 [12,52]	53-105,1	72,5
BMI (kg/m^2)	24,70 [2,45]	20,90-28,02	25,1

Supplementary Table 3, overview of the successful microbiota composition determination

Supplementary table 3, overview of the successful microbiota composition determination in ileostomy samples collected during the study per enrolled subject.

**Subject**	**No. of successful samples (max 27)**	**Success rate (%)**	**Averaged DNA concentration (ng/ul)**
VOL01	24	88.9	16.32
VOL02	26	96.3	29.31
VOL03	26	96.3	16.19
VOL04	26	96.3	18.70
VOL05	27	10.0	8.72
VOL06	26	96.3	20.41
VOL07	27	10.0	5.15
VOL08	27	10.0	14.27
VOL09	21	77.8	12.71
VOL10	15	55.6	15.32
VOL11	26	96.3	20.88
VOL12	27	10.0	133.17
VOL13	13	48.1	0.23
VOL14	25	92.6	13.39
VOL15	27	10.0	12.72
VOL16	27	10.0	10.66

Supplementary Table 4, successful samples and success rate per trial phase

Supplementary table 4, successful samples and success rate per trial phase. The two wash out periods were summed.

Trial phase	No. of successful samples	Success rate (%)
Run in	43	89.6%
Yogurt	86	89.6%
Wash out	56	87.5%
Placebo	87	90.6%
L. rhamnosus	92	95.8%
Run out	26	81.3%

Supplementary Table 5, differential abundance analysis, species

Supplementary table 5: differential abundance analysis of species, analysed by EdgeR. Prevalence: percentage of subjects with average relative abundance of the indicated species higher than 1%. Abundance: average relative abundance of the indicated species in the subjects in which it was detected.

**Rhamno vs Placebo**				
Species	Fold change	FDR *p*	Prevalence	Abundance
Ambiguous_taxa-Enterococcus	-3.94	4.86E-12	13%	.005
Clostridiales_Clostridiales	1.19	2.85E-02	0%	N/A
**Yogurt vs Placebo**				
Ambiguous_taxa-Enterococcus	-3.98	1.77E-11	7%	2.33E-03
Enterococcus faecalis_Enterococcus	3.13	4.70E-07	0%	9.32E-04
uncultured Gemella sp._Bacillales	-2.76	1.29E-06	0%	5.22E-04
uncultured Streptococcus sp._Streptococcus*	-1.93	3.38E-05	27%	6.65E-03
Ambiguous_taxa-Actinomyces	-1.48	5.20E-03	0%	1.08E-03
Streptococcus gordonii_Streptococcus	-1.55	1.84E-02	0%	1.36E-04
uncultured bacterium-Granulicatella	-1.66	1.84E-02	0%	1.90E-04
uncultured organism-Haemophilus	-1.84	1.85E-02	27%	1.44E-02
uncultured bacterium-Bacillales	-1.42	1.99E-02	0%	N/A
uncultured bacterium-Corynebacterium	-1.58	2.77E-02	0%	1.87E-04
uncultured organism-Ruminococcus	1.75	3.15E-02	0%	8.61E-05
Ambiguous_taxa-Romboutsia	-1.91	4.02E-02	27%	1.01E-02
uncultured bacterium-Atopobium	2.07	4.02E-02	0%	9.10E-05
uncultured bacterium-Aggregatibacter	-1.23	4.02E-02	0%	1.71E-04
Granulicatella sp. oral clone ASCG05_Granulicatella	-1.05	4.04E-02	0%	7.21E-04
Bacteroides sp. 3_2_5_Bacteroides	2.13	4.24E-02	27%	6.64E-03
uncultured bacterium-Lachnoanaerobaculum	-1.32	4.29E-02	0%	1.22E-04

Supplementary Table 6, differential abundance analysis, pathways

Supplementary table 6: differential abundance analysis of pathways of FMM, analysed by EdgeR. Subject ID used as secondary blocking factor. No FMM pathway changes were detected upon consumption of Yogurt relative to the Placebo.

**Rhamno vs Placebo**		
Pathway	Fold change	FDR p-val
L-rhamnose degradation I	4.8252	.043447
**Rhamno vs Yogurt**		
Hexitol fermentation to lactate, formate, ethanol and acetate	2.7772	.000497
Superpathway of fucose and rhamnose degradation	3.1322	.001466
L-rhamnose degradation I	3.324	.001466
Acetyl-CoA fermentation to butanoate II	2.5852	.001466
Homolactic fermentation	1.2499	.008906
Stachyose degradation	1.7456	.011369
Superpathway of hexitol degradation (bacteria)	1.7412	.011952
Glycolysis IV (plant cytosol)	1.3102	.046822

Supplementary References

1. van Wijck K, van Eijk HM, Buurman WA, et al. Novel analytical approach to a multi-sugar whole gut permeability assay. J Chromatogr B Analyt Technol Biomed Life Sci 2011;879:2794-801.
2. van Wijck K, Verlinden TJ, van Eijk HM, et al. Novel multi-sugar assay for site-specific gastrointestinal permeability analysis: a randomized controlled crossover trial. Clin Nutr 2013;32:245-51.
3. Garcia-Villalba R, Gimenez-Bastida JA, Garcia-Conesa MT, et al. Alternative method for gas chromatography-mass spectrometry analysis of short-chain fatty acids in faecal samples. J Sep Sci 2012;35:1906-13.
