# Supplementary material for: Endogenous small intestinal microbiome determinants of transient colonisation efficiency by bacteria from fermented dairy products: a randomised controlled trial
Source: Microbiome. 2023 Mar 7;11:43. doi: 10.1186/s40168-023-01491-4 (PMC9990280; doi:10.1186/s40168-023-01491-4)
Supplement: Supplementary file 5 — Additional file 4. CONSORT flow diagram. [file 40168_2023_1491_MOESM4_ESM.doc]

**
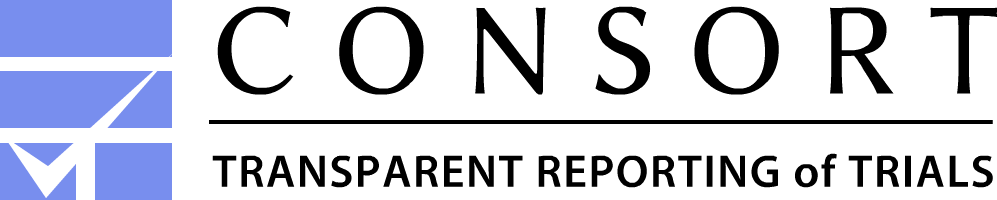
**

**CONSORT 2010 Flow Diagram**

**Enrollment**

Ileostomy patients received an information letter about the study (n= 108)

Excluded (n=2)

♦  Not meeting inclusion criteria, more than 15cm of ileum had been removed (n=2)

Screening of ileostomy patients (n=18)

Allocated *L. rhamnosus* (n=5)

♦ Received allocated intervention (n=5)

Allocated Yogurt (n=7)

♦ Received allocated intervention (n=7)

**Allocation intervention 1**

Allocated Placebo (n=4)

♦ Received allocated intervention (n=4)

Randomization of the interventions order (n=16)

2 weeks wash-out

Cross-over

**Analysis**

**Follow-Up**

**Allocation intervention 2**

Allocated *L. rhamnosus* (n=6)

♦ Received allocated intervention (n=6)

Allocated Yogurt (n=5)

♦ Received allocated intervention (n=5)

Allocated Placebo (n=5)

♦ Received allocated intervention (n=5)

2 weeks wash-out

Cross-over

**Allocation intervention 3**

Lost to follow-up (n=0)

Discontinued intervention (n=0)

Analysed (n=15)
♦ Excluded from analysis, Kock pouch ileostomy patient (n=1)

Allocated Placebo (n=7)

♦ Received allocated intervention (n=7)

Allocated *L. rhamnosus* (n=5)

♦ Received allocated intervention (n=5)

Allocated Yogurt (n=4)

♦ Received allocated intervention (n=4)

2 weeks run-out
